# An ensemble data assimilation modeling system for operational outdoor microalgae growth forecasting

**DOI:** 10.1002/bit.28272

**Published:** 2022-11-06

**Authors:** Hongxiang Yan, Mark S. Wigmosta, Michael H. Huesemann, Ning Sun, Song Gao

**Affiliations:** ^1^ Pacific Northwest National Laboratory Richland Washington USA; ^2^ Department of Civil and Environmental Engineering University of Washington Seattle Washington USA; ^3^ Marine and Coastal Research Laboratory, Pacific Northwest National Laboratory Sequim Washington USA

**Keywords:** biomass forecasting, *Chlorella sorokiniana*, data assimilation, Huesemann Algae Biomass Growth Model, particle filter

## Abstract

Microalgae have received increasing attention as a potential feedstock for biofuel or biobased products. Forecasting the microalgae growth is beneficial for managers in planning pond operations and harvesting decisions. This study proposed a biomass forecasting system comprised of the Huesemann Algae Biomass Growth Model (BGM), the Modular Aquatic Simulation System in Two Dimensions (MASS2), ensemble data assimilation (DA), and numerical weather prediction Global Ensemble Forecast System (GEFS) ensemble meteorological forecasts. The novelty of this study is to seek the use of ensemble DA to improve both BGM and MASS2 model initial conditions with the assimilation of biomass and water temperature measurements and consequently improve short‐term biomass forecasting skills. This study introduces the theory behind the proposed integrated biomass forecasting system, with an application undertaken in pseudo‐real‐time in three outdoor ponds cultured with *Chlorella sorokiniana* in Delhi, California, United States. Results from all three case studies demonstrate that the biomass forecasting system improved the short‐term (i.e., 7‐day) biomass forecasting skills by about 60% on average, comparing to forecasts without using the ensemble DA method. Given the satisfactory performances achieved in this study, it is probable that the integrated BGM‐MASS2‐DA forecasting system can be used operationally to inform managers in making pond operation and harvesting planning decisions.

## Graphical abstract

The four components of the proposed biomass forecasting system: (1) two models: biomass growth model (BGM) to model microalgae growth and MASS2 hydrodynamic model to model water temperature. (2) Two measurements: automatically/manually measured pond water temperature and biomass density. (3) Numerical weather forecasts: state‐of‐the‐art sequentially updated 7‐day weather forecasts. (4) Ensemble data assimilation algorithm: particle filtering with bias estimation to assimilate the measurements into the models to enhance biomass forecasts.

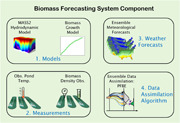

**Abbreviations**
BAT: biomass assessment tool
BGM: Huesemann Algae Biomass Growth Model
CIMIS: California Irrigation Management Information System
DA: data assimilation
DI: direct insertion
GEFS: Global Ensemble Forecast System
MASS2: Modular Aquatic Simulation System in Two Dimensions
MCRPS: mean continuous ranked probability score
NWP: numerical weather prediction
OD_750_
: optical density at 750 nm
OL: open loop
PAR: photosynthetically active radiation
PDF: probability density function
PF: particle filter
PFBE: particle filter with bias estimation
RMSE: root‐mean‐squared‐error
USDOE: US Department of Energy

## 1 INTRODUCTION

Microalgae biofuel has received growing attention in the last decade due to the increasing demands for alternative energy sources, national energy security, and global carbon emission reduction (Wigmosta et al., 2011). In December 2007, the US Congress enacted the Energy Independence and Security Act which recognized microalgae holds great promise as a supplemental domestic biofuel feedstock (USDOE, 2010). To achieve economical and sustainable large‐scale microalgae biofuel production, there is an emerging need to identify strains that exhibit high annual biomass productivities at different geographic locations and in different seasons (Gao et al., 2018; M. H. Huesemann et al., 2013; Xu et al., 2019).

In the recent US Department of Energy (USDOE) National Alliance for Advanced Biofuels and Bioproducts consortium project, *Chlorella sorokiniana* (USDOE 1412) was identified as one of the most productive strains with a maximum specific growth rate of about 5.9 day^−1^ under optimal conditions (Huesemann et al., 2018). To extrapolate this finding in laboratory cultures to outdoor pond cultures, Pacific Northwest National Laboratory developed the biomass assessment tool (BAT) (Coleman et al., 2014; Wigmosta et al., 2011; Xu et al., 2019) that provides a national assessment of biomass production by considering local climate conditions, water resources, sources of CO_2_, and land prices (Venteris et al., 2012, 2013). In the BAT, Huesemann et al. (2016) developed the Huesemann Algae Biomass Growth Model (BGM) to predict biomass productivity in outdoor ponds under nutrient‐replete and well‐mixed operating conditions. Because light and water temperature are the primary controlling abiotic factors of biomass productivity, the *Chlorella sorokiniana*‐specific growth parameters for BGM were determined over a range of temperature and light intensities in laboratory cultures. In the recent USDOE Regional Algal Feedstock Testbed project, the BGM was validated against observed outdoor pond biomass productivity in different seasons in the southwest United States under diurnally fluctuating light and water temperatures and has proved to be a reliable tool for national biomass productivity assessment (Gao et al., 2018; Khawam, Waller, Gao, Edmundson, Huesemann, et al., 2019; Khawam, Waller, Gao, Edmundson, Wigmosta, et al., 2019). To evaluate site‐specific biomass productivity and guide site selection for commercial‐scale microalgae production over the nation, the hydrodynamic Modular Aquatic Simulation System in Two Dimensions (MASS2; Perkins & Richmond, 2004) within the BAT is used to predict pond water temperature for the BGM using the measured or reanalysis site‐specific meteorological data.

Despite these significant modeling efforts over the past years, major challenges still exist in optimizing large‐scale outdoor microalgae culture operations and harvesting planning (Christenson & Sims, 2011; Mathimani & Mallick, 2018; Pienkos & Darzins, 2009). In outdoor open ponds, optimizing pond operations to achieve the highest biomass productivity requires an optimal selection of harvesting time and dilution rate (ratio of the volume of harvested media to the total volume), which is difficult to schedule in advance and entirely depends on an operator's expert judgment at present. Further, the delivery of photosynthetically active radiation (PAR) in terms of solar radiation and pond water temperature is associated with climate variability that reflects sub‐hourly variance. The large interface between the air and liquid culture also increases the chances of biological contamination, leading to loss or failure of biomass productivity (Gao et al., 2018; Khawam, Waller, Gao, Edmundson, Huesemann, et al., 2019; Wang et al., 2013). Besides optimizing pond operations, optimizing biomass harvesting is also an economical key for advancing commercial‐scale biofuel production as the harvesting costs can occupy about 30% of the total capital investment for biofuel (Jankowska et al., 2017; Milledge & Heaven, 2013). The biofuel logistic chain consists of a range of activities that include harvesting, bailing, storing, drying, and transportation to a downstream biorefinery. Thus, optimizing harvesting planning (e.g., harvesting time, labor, amount of biomass, storage facilities) is critical to enhancing the biofuel logistic chain such as biomass storing and transporting (Ba et al., 2016; De Meyer et al., 2014).

A potential solution to optimize both pond operations and harvesting planning is to forecast microalgae growth days to weeks ahead so that operators can determine when are the best harvesting time and logistics managers can optimize harvesting planning (e.g., resource allocation, labor, storage) with days to weeks preparation time. For example, if the biomass productivity is forecasted to decline in the next few days because the weather is expected to get colder, operators will know now it is the time to harvest to avoid biomass loss or reduce resource inputs; if clients request one‐ton of biomass, and the forecasts suggest there will be a 20% chance of one‐ton biomass in 3 days and 98% chance of one‐ton biomass in 5 days, based on the weather forecast, the manager will schedule the harvesting activities, allocate resources, and optimize the supply chain to 5 days.

The majority of the previous microalgae growth prediction scenario‐based studies focused on long‐term average biomass yield under fixed operating conditions (Sun et al., 2020; Xu et al., 2019); short‐range (days to weeks) microalgae growth forecasting on a local‐scale is relatively new but has received increasing attention in both academia and industry (Gao et al., 2022; Yan et al., 2021). In outdoor open ponds, two primary factors control microalgae growth forecasting skills: the skill of weather forecasts in the coming days to weeks and the knowledge of initial conditions that are the biomass and pond water temperature conditions existing at the time the forecast is made (Page et al., 2018; Yan et al., 2021). Note that “skill” is a terminology used to describe any measure of the accuracy and/or degree of association of predictions to observations.

Advances in numerical weather prediction (NWP), inexpensive biomass density measurements in terms of optical density at 750 nm (OD_750_), and automatic water temperature measurements make effective real‐time biomass forecasts feasible (Alley et al., 2019; Van Wagenen et al., 2012; Yan et al., 2021). For example, depending on the growth conditions, *Chlorella sorokiniana* can have a high biomass density and be ready for harvesting in less than 7 days (Huesemann et al., 2016); and today's NWP is sufficient to provide reliable weather forecasts in the coming 3–7 days with a forecasting skill of more than 95% and 70%, respectively (Bauer et al., 2015). It is known that the biomass model simulations contain inherent uncertainties that arise from the input data and model parameter uncertainties. To reduce water temperature and biomass initial condition uncertainties (i.e., model estimates at the time the forecast is made), ensemble data assimilation (DA), a technique that optimally merges information from model simulation and measurements (e.g., OD_750_) with appropriate uncertainty modeling, is key to improving initial condition characterization and, consequently, forecasting skill (Abbaszadeh et al., 2018; Yan & Moradkhani, 2016; Yan et al., 2015, 2017; Zarekarizi et al., 2021). The ensemble DA technique marks a major milestone in the forecasting community and is now operationally implemented in NWP and flood forecasting around the globe (Bauer et al., 2015; Cloke & Pappenberger, 2009; Habert et al., 2014; Pappenberger et al., 2011).

Based on the above discussion, the objective of this study is to propose and examine an integrated modeling system with the use of ensemble DA for operational microalgae growth forecasting. Specifically, the integrated biomass forecasting system consists of state‐of‐the‐art NWP products, coupled MASS2‐BGM model, and the ensemble DA method. We aim to determine the effectiveness of forecasting biomass by assimilating biomass density and water temperature measurements into BAT's BGM and MASS2 models.

The rest of the paper is organized as follows: Section 2 describes the methods and experimental design, which includes the integrated BGM‐MASS2‐DA biomass forecasting system, the model descriptions, the microorganisms and media, and the ensemble DA algorithm. Section 3 presents the biomass simulation and forecasting results using three outdoor pond case studies located in Delhi, California, United States. Finally, Section 4 concludes the paper and provides research directions for advancing operational biomass forecasting.

## 2 METHODS

In this section, we describe the integrated BGM‐MASS2‐DA biomass forecasting system and experiment design, followed by more detailed descriptions of the components and data sources.

### 2.1 The integrated biomass forecasting system

**Figure 1:**
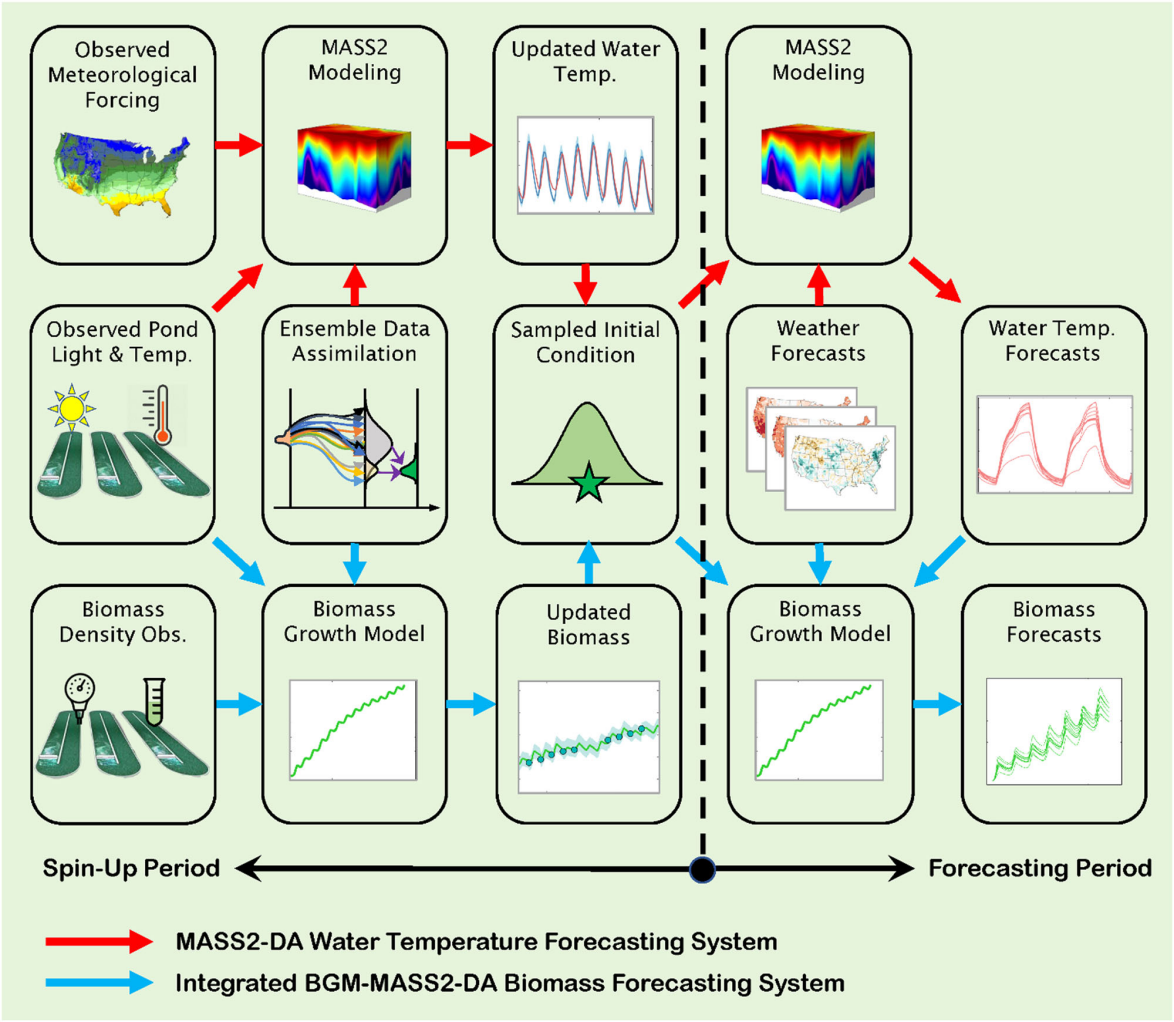
Framework of the MASS2‐DA water temperature forecasting system by assimilating water temperature observation and integrated BGM‐MASS2‐DA biomass forecasting system by assimilating both water temperature and biomass observations. Before each forecasting date (spin‐up period), the ensemble data assimilation (DA) technique is used to assimilate water temperature and biomass observations to update MASS2 and BGM initial states and characterize uncertainties (in terms of a probability density function). At each forecasting date, updated states or called initial conditions shown as “green star” are sampled from the probability distributions and used to generate the 7‐day ensemble forecasts.

The integrated BGM‐MASS2‐DA biomass forecasting system is illustrated in Figure 1. The integrated forecasting system includes both spin‐up and forecasting periods that are separated by the forecast initialization time. During the spin‐up period, two ensemble DA systems are performed in parallel until the forecast initialization time: (1) the MASS2‐DA system to assimilate observed pond water temperature to improve pond water temperature initial conditions and (2) the BGM‐DA system to assimilate observed OD_750_ to improve pond biomass initial conditions. In the BGM‐DA system, the observed light and pond water temperature is first used to drive the BGM and generate biomass simulations. Whenever biomass observations become available, the ensemble DA technique is used to assimilate observations to improve the biomass simulations and quantify the initial condition uncertainty at each forecasting initialization time. The initial condition uncertainty is quantified through a probability density function (PDF) derived from the updated ensemble biomass states. Then at each forecast initialization time, an initial condition sampled from the PDF is input to the BGM for forecasts. A similar procedure is used in the MASS2‐DA and hence will not be repeated here. During the forecasting period, the NWP 7‐day meteorological forecasts are first used to drive MASS2 with the updated initial pond water temperature to generate the 7‐day water temperature forecasts. Then, the 7‐day water temperature forecasts from MASS2 and the 7‐day solar forecasts from NWP are used to drive BGM with the updated initial biomass condition to generate 7‐day biomass forecasts.

### 2.2 Microorganisms and media

*Chlorella sorokiniana* (USDOE 1412) was originally isolated from surface water in Texas by Dr. Juergen Polle from Brooklyn College and grown at pH 7 in a freshwater BG‐11 medium containing 0.66 mM PO_4_ and 17.6 mM NO_3_ (Andersen, 2005; Huesemann et al., 2013; Neofotis et al., 2016). *Chlorella sorokiniana* was cultured in batch mode in three outdoor raceway ponds (30 cm depth) in nutrient‐replete BG‐11 medium, maintained at pH 7 via intermittent CO_2_ sparging, in Delhi, California, United States, from late June to early July 2015. Makeup water was added daily to compensate for evaporation loss and maintain the 30 cm pond depth. Before the outdoor pond experiments, the maximum specific growth rate of *C. sorokiniana*, in addition to the loss rate in the dark, had been determined experimentally as a function of both temperature and light intensity in laboratory cultures (Huesemann et al., 2016).

### 2.3 Biomass assessment tool

The BAT is an integrative modeling platform that includes the physics‐based BGM and MASS2 models, geographic information systems, and high‐resolution spatial and temporal analysis (e.g., water stress, land resource) to assess the amounts and geographic distribution of potential algal biofuel production in the United States (Wigmosta et al., 2011). In this forecasting experiment, we only used the BGM and MASS2 models within the BAT. As both BGM and MASS2 models have been described in a sufficient body of prior literature (Coleman et al., 2014; Gao et al., 2018; Huesemann et al., 2016, 2017; Khawam, Waller, Gao, Edmundson, Huesemann, et al., 2019; Perkins & Richmond, 2004; Venteris et al., 2014; Wigmosta et al., 2011), only a brief description is presented here. For details, the readers are referred to Huesemann et al. (2016) for BGM and Perkins and Richmond (2004) for MASS2.

#### 2.3.1 The BGM model

The BGM uses incident light intensity (i.e., PAR) and algae culture temperature to determine biomass growth rate in the algae culture. Because the strain was parameterized in sterile nutrient‐replete BG‐11 medium at pH 7.0 (Huesemann et al., 2016), the BGM is applicable to pond cultures that are also grown under these conditions, that is, nutrient‐replete, pH near 7.0, and no other growth inhibitors (e.g., invasive species). In the BGM modeling, the outdoor pond volume is discretized vertically into many equal volume layers. In each layer, the biomass concentration B is assumed to increase exponentially in a time interval ∆t as

(1) $$B\left(t+∆t\right)=B\left(t\right)e^{\mu ∆t},$$

where μ is the biomass growth rate (day^−1^) in the respective layer. The biomass growth rate μ, affected by both water temperature and light intensity, is expressed as

(2) μ=f(T,I),

where T is the pond water temperature and I is the light intensity. As each microalgae strain has a unique response to the combination of light and temperature, the response function f(T,I) is strain‐specific and needs to be experimentally determined before running the model.

According to the Beer–Lambert Law (Ingle & Crouch, 1988), for a given biomass concentration, light intensity (PAR, 400–700 nm) attenuates as a function of light penetration distance as

(3) $$I\left(z\right)=I_{0}e^{-k_{a}\mathrm{Bz}},$$

where I(z) is the light intensity at depth z, $I_{0}$ is the light intensity at the culture surface, $k_{a}$ is the biomass light absorption coefficient for the entire PAR range (400–700 nm), and B is the biomass concentration. To account for possible light scattering in dense biomass cultures, the BGM used an experimentally determined scatter‐corrected biomass light attenuation coefficient ($k_{\text{sca}}$), following the method from Suh and Lee (2003), for the prediction of light intensity as a function of light penetration distance and biomass concentration:

(4) $$k_{\text{sca}}=k_{a}\frac{K_{B}}{K_{B}+B}\frac{K_{z}}{K_{z}+z},$$

where $K_{B}$ and $K_{z}$ are the light scattering coefficients associated with biomass concentration B and light penetration distance z, respectively. Measurement of $k_{\text{sca}}$ is done in a white translucent container mixed from below with a magnetic stirrer and illuminated from above with a multi‐color LED panel simulating sunlight at ca. 2000 μmol m^−^^2^ s^−1^. For details, readers are referred to Huesemann et al. (2016).

The outdoor pond will lose biomass at night through dark respiration. The rate of biomass loss $\mu_{\text{dark}}$ in the nighttime is estimated as a function of pond water temperature T and the average light intensity $I_{\text{avg}}$ during the preceding day as

(5) $$\mu_{\text{dark}}=f\left(T,I_{\text{avg}}\right).$$

The $I_{\text{avg}}$ is estimated by averaging the depth‐integrated light attenuation profiles for each time interval ∆t over the entire day preceding the night. Like the biomass growth rate μ, the biomass loss rate $\mu_{\text{dark}}$ is also determined by laboratory experiments.

#### 2.3.2 The MASS2 model

The MASS2 is an unsteady flow, a two‐dimensional model that simulates hydrodynamics and water quality in ponds, rivers, and estuaries. The MASS2 used a structured multiblock, curvilinear computational mesh to discretize outdoor pond volume and a finite volume method to solve the energy conservation equation as (Patankar, 1980):

(6) $$h_{1}h_{2}\frac{\partial \left(\mathrm{dT}\right)}{\partial t}+\frac{\partial \left(h_{2}\mathrm{dUT}\right)}{\partial \xi }+\frac{\partial \left(h_{1}\mathrm{dVT}\right)}{\partial \eta }=\frac{\partial }{\partial \xi }\left(h_{2}\frac{\varepsilon_{1}}{h_{1}}d\frac{\partial T}{\partial \xi }\right)+\frac{\partial }{\partial \eta }\left(h_{1}\frac{\varepsilon_{2}}{h_{2}}d\frac{\partial T}{\partial \eta }\right)+\frac{h_{1}h_{2}H}{\rho c_{v}},$$

where $h_{1}$ and $h_{1}$ are curvilinear grid metric coefficients in the ξ and η directions, U and V are the depth‐averaged velocities in the ξ and η directions, d is the water depth, $\varepsilon_{1}$ and $\varepsilon_{2}$ are the turbulent eddy diffusion coefficients in the ξ and η directions, H is the net surface heat flux at the water surface, ρ is the water density, and $c_{v}$ is the specific heat of the water. The net surface heat flux is estimated as

(7) $$H=H_{s}+H_{a}-(H_{b}+H_{e}+H_{c}),$$

where $H_{s}$ is the net solar shortwave radiation, $H_{a}$ is the net atmospheric longwave radiation, $H_{b}$ is the longwave back radiation, $H_{e}$ is the latent heat flux, and $H_{c}$ is the sensible heat flux. The MASS2 model parameters (e.g., bed depth, conduction rate) were adjusted to represent the elevated outdoor pond conditions in Delhi, California.

### 2.4 Data sources

In the outdoor pond microalgae growth experiment, the hourly light intensity and pond water temperature data were automatically measured on‐site for the microalgae growth periods from June 30 to July 13, 2015. The pond biomass concentrations were manually measured as OD_750_ once or twice a day. In the MASS2 modeling spin‐up periods, local hourly meteorological forcing data at Delhi, California, including air temperature, wind speed, dew point temperature, air pressure, and shortwave radiation, were acquired from nearby Station #206 of the California Irrigation Management Information System (CIMIS, https://cimis.water.ca.gov/).

In the forecasting periods, the hourly forecasts of meteorological data (air temperature, wind speed, specific humidity, air pressure, and shortwave radiation) at lead times of 7 days were acquired from the recently released 2nd‐Generation National Oceanic and Atmospheric Administration global ensemble reforecast data set at 1° × 1° grid cell (Hamill et al., 2019) that is statistically consistent with the currently operational National Center for Environmental Prediction Global Ensemble Forecast System (GEFS) (Hamill et al., 2013). In the GEFS reforecasts, perturbations are made in initial atmospheric conditions to generate ensemble members of the forecast. The GEFS reforecast was generated once daily at 00 Coordinated Universal Time and contained 11 ensemble members. In this study, the 7‐day GEFS reforecasts were downloaded for the microalgae growth periods from June 30 to July 5, 2015 (a total of six forecast cycles).

### 2.5 Ensemble data assimilation and forecast evaluation

The ensemble DA system is used to update model simulations by assimilating observations with appropriate uncertainty quantification and consequently, improve initial condition characterization for forecasting. Among the various ensemble DA methods, the particle filter (PF) has advantages in handling nonlinear, non‐Gaussian systems, preserving mass and energy balance in hydrodynamic and microalgae growth modeling, and providing a more complete representation of the state posterior distribution (Abbaszadeh et al., 2018; Ahmadalipour et al., 2017; Yan et al., 2015, 2018; Zarekarizi et al., 2021).

The PF is a class of sequential Monte Carlo methods used to update the model states and/or parameters when new observations become available. Ensemble model predictions are generated by different runs simulated by different meteorological forcing data (e.g., the perturbed forcing). The collection of individual model estimates, called particles, is referred to as the prior distribution of the model state. These particles are advanced in parallel. At the time step when an observation is available, the PF algorithm assigns lower weights for the particles that diverge from observations and higher weights for the particles that are close to observations. A resampling operation is then used to remove the particles with low weights and duplicate particles with high weights. The collection of resampled particles is referred to as the posterior distribution of the model state.

#### 2.5.1 Particle filter

The differential equations that describe the nonlinear microalgae growth dynamics are described as follows:

(8) $$x_{t}=f\left(x_{t-1},u_{t},\theta \right)+\omega_{t},\omega_{t}\sim N\left(0,Q_{t}\right),$$

(9) $$y_{t}=h\left(x_{t}\right)+v_{t},v_{t}\sim N\left(0,R_{t}\right),$$

where $x_{t}\in R^{+}$ is the uncertain biomass concentration state variable at time t, $u_{t}\in {\mathbb{R}}^{m}$ is a vector of uncertain hydrometeorological forcing data, $\theta \in {\mathbb{R}}^{n}$ is a vector of microalgae growth model parameters, $y_{t}\in R^{+}$ represents biomass concentration measured as OD_750_, $\omega_{t}$ and $v_{t}$ represent the model and measurement errors. More often, $\omega_{t}$ and $v_{t}$ are assumed to be independent and follow the white noises with mean zero and variances $Q_{t}$ and $R_{t}$, respectively. The nonlinear function f(∙) relates the state at the previous time step t−1 to the state at the current time step t, the function h(∙) relates the state $x_{t}$ to the measurement $y_{t}$. Similar differential equations are also applied for pond water temperature dynamics and hence will not be repeated here.

Based on the Bayes' Law, the posterior distribution of the state variable at time t is estimated as follows:

(10) $$p\left(x_{t}|y_{1:t}\right)=\frac{p\left(y_{t}|x_{t}\right)p\left(x_{t}|y_{1:t-1}\right)}{p(y_{t}|y_{1:t-1})}=\frac{p\left(y_{t}|x_{t}\right)p\left(x_{t}|y_{1:t-1}\right)}{\int p\left(y_{t}|x_{t}\right)p\left(x_{t}|y_{1:t-1}\right)dx_{t}},$$

(11) $$p\left(x_{t}|y_{1:t-1}\right)=\int p\left(x_{t}|x_{t-1}\right)p\left(x_{t-1}|y_{1:t-1}\right)dx_{t-1},$$

where $p\left(y_{t}|x_{t}\right)$ is the likelihood at time t, $p\left(x_{t}|y_{1:t-1}\right)$ is the prior distribution, and $p(y_{t}|y_{1:t-1})$ is the normalization factor. Analytical solution of Equation (10) is only feasible for special cases such as a linear system with Gaussian noise, for practical reasons, the posterior distribution is approximated using a set of particles with associated weights as follows:

(12) $$p\left(x_{t}|y_{1:t}\right)\approx \sum_{i=1}^{N}w^{i+}\delta \left(x_{t}-x_{t}^{i}\right),$$

where $w^{i+}$ is the posterior weight of the ith particle, δ is the Dirac delta function, and N is the particle number. The posterior weight is estimated as follows:

(13) $$w^{i+}=\frac{w^{i-}p(y_{t}|x_{t}^{i})}{\sum_{i=1}^{N}w^{i-}p(y_{t}|x_{t}^{i})},$$

where $w^{i-}$ is the prior weight of the ith particle, the likelihood $p(y_{t}|x_{t}^{i})$ is estimated using the Gaussian distribution as follows:

(14) $$p\left(y_{t},|,x_{t}^{i}\right)=\frac{1}{\sqrt{2\pi R_{t}}}e^{-\frac{{\left[y_{t}-h(x_{t}^{i})\right]}^{2}}{2R_{t}}.}$$

A resampling operation is necessary to obtain approximate particles from $p\left(x_{t}|y_{1:t}\right)$. At each time step, we resample the particles with a probability greater than the uniform probability (Moradkhani, Hsu, et al., 2005). After the resampling, particles with higher weights are retained and particles with lower weights are eliminated; all particle weights are then set to 1/N.

#### 2.5.2 Particle filter with bias estimation

In the standard PF method as described above, a fundamental assumption is that the measurement errors ($v_{t}$) as well as the model simulation errors ($\omega_{t}$) are both unbiased. Measurement errors are related to representativeness errors, instrumental inaccuracies, and remote‐sensing retrieval errors (Van Wagenen et al., 2012). In general, after the quality control process, measurement errors are largely unbiased and validated for the random error assumption (De Lannoy, Reichle, et al., 2007). Model simulations, on the contrary, are often biased caused by uncertain hydrometeorological forcing, imperfect model structure, and parameter estimations. This is especially the case in outdoor pond operating culture systems because the growth and loss rate parameters were previously determined in laboratory cultures with a few pairs of light intensity and water temperature (Gao et al., 2018; Khawam, Waller, Gao, Edmundson, Huesemann, et al., 2019); differences in pH and nutrients between the laboratory culture and outdoor pond culture are also likely. As a result, model simulation errors often contain both random error and systematic error components, and the latter could vary with time (De Lannoy, Houser, et al., 2007).

To correct the biased microalgae simulations in PF, Yan et al. (2021) developed the particle filter with bias estimation (PFBE) method. We added a time‐varying model bias term in the dynamic system and the bias term will be updated at the time step with the assimilation of new observations. Therefore, with more frequent observations, the model bias will be better tuned to reflect the model systematic error. When estimating the model posterior distributions, the difference between the resampled model states and the resampled bias states will be used and reflect the model random error.

A time‐varying model bias term $b_{t}$ is added in the nonlinear microalgae growth model as

(15) $$x_{t}^{\prime }=f\left(x_{t-1},u_{t},\theta ,b_{t}\right)+\omega_{t},\omega_{t}\sim N\left(0,Q_{t}\right),$$

where $x_{t}^{\prime }$ is the unbiased model simulation and the model bias $b_{t}$ is modeled using a forward model operator as

(16) $$b_{t}=g\left(b_{t-1}\right)$$

the posterior distribution from Equation (12) is approximated as

(17) $$p\left(x_{t},b_{t}|y_{1:t}\right)\approx \sum_{i=1}^{N}w^{i+}\delta \left(x_{t}-x_{t}^{i},b_{t}-b_{t}^{i}\right)$$

and the weight estimation is estimated as

(18) $$w^{i+}=\frac{w^{i-}p(y_{t}|x_{t}^{i},b_{t}^{i})}{\sum_{i=1}^{N}w^{i-}p(y_{t}|x_{t}^{i},b_{t}^{i})}.$$

In addition to resampling of the state variables, resampling in the bias space can also be carried out by using the forward operator in Equation (16) as the proposal density in the standard PF algorithm. Thus, this joint state‐bias estimation is the same as the joint state‐parameter PF implementation by Moradkhani, Hsu, et al. (2005). Despite resampling can reduce particle degeneracy problem that indicates ensemble collapse to a single particle, it may also lead to sample impoverishment or loss of diversity among particles that many particles having higher weights being selected many times. To maintain diversity throughout the bias ensemble, a small error term is added to the bias after each resampling step following Moradkhani, Sorooshian,  et al. (2005).

With the absence of an explicit form for the bias forward operator in microalgae dynamics (Chepurin et al., 2005), an adaptive linear model is used to propagate the bias estimate in time between two successive assimilation updates as

(19) $$b_{t}^{i-}=\beta_{t}b_{t-n}^{i+}+\varepsilon_{t}^{i},\varepsilon_{t}^{i}\sim N\left(0,s\text{Var}(b_{t-n}^{i-})\right),$$

(20) $$\beta_{t}=\frac{\sum_{i=1}^{N}x_{t}^{i}/N-h^{-1}\left(y_{t}\right)}{\sum_{i=1}^{N}x_{t-n}^{i}/N-h^{-1}\left(y_{t-n}\right)},$$

where $\text{Var}(b_{t-n}^{i-})$ is the variance of the prior bias at the time step t−n, s is a small tuning parameter with a typical range between 0.005 and 0.025 (Moradkhani et al., 2012; Yan et al., 2015), and n is the time lag between two successive measurements. The adaptive linear model assumption is respectively valid if the measurement can approximate the true state through the inverse function $h^{-1}\left(∙\right)$. This assumption is more appropriate for small measurement errors and linear operation h such as the biomass concentration that is measured as the reliable OD_750_ (Huesemann et al., 2016; Van Wagenen et al., 2012).

In the PFBE, the adaptive linear model is used for modeling bias evolution because biomass concentration was measured at a daily time step while the BGM was simulated at an hourly step. This inconsistency between the observation scale and modeling scale leads to model bias at time t that could deviate largely from the previous bias at time t−n (Gao et al., 2018; Huesemann et al., 2016). To achieve optimal DA performances, biomass concentration is better to be measured by matching the modeling timestep. In such a case (i.e., hourly measurements by using automatic biomass sensors), a persistence bias forward model ($b_{t}^{i-}=b_{t-1}^{i+}+\varepsilon_{t}^{i}$) is preferred to relax the implicit assumption within the adaptive linear model because bias evolves more slowly in small time steps (De Lannoy, Reichle, et al., 2007; Dee & Da Silva, 1998; Keppenne et al., 2005). A balance between biomass measurement frequency and DA performance needs further study. The resampling of the bias is decoupled from the computation of the “bias‐blind” estimate of the biomass $x_{t}$ in Equation (8). The bias term does not feed back into the BGM modeling but rather corrects the output (Friedland, 1969). Thus, the mass and energy balance in the BGM is still preserved. In the forecasting mode, we used the estimated bias posteriors in the forecasting initialization date to correct the BGM 7‐day biomass forecasts driven by GEFS reforecasts.

#### 2.5.3 Error models

In the BGM‐DA spin‐up simulations, to account for uncertainties in measured light intensity (i.e., PAR) and pond water temperature due to sensor errors, PAR was assumed to be heteroscedastic with 25% multiplicative normal error, and the water temperature was assumed to be homoscedastic with 2°C additive normal error. Cross‐correlation with perturbations in PAR and water temperature was set to be 0.48. BGM model structure error was assumed to be normally distributed with SDs equal to 15% of BGM simulation values. In the MASS2‐DA spin‐up simulations, to account for uncertainties in the CIMIS forcing data, both air and dewpoint temperature was assumed to be homoscedastic with 4°C additive normal errors; shortwave radiation, wind speed, and surface pressure assumed to be heteroscedastic with 50% multiplicative normal errors. Cross‐correlations in air and dewpoint temperature, air temperature, and shortwave radiation were set to be 0.53 and 0.44, respectively. MASS2 model structure error was assumed to be normally distributed with an SD equal to 1°C. The tuning parameter s in Equation (19) was set to be 0.005. The form and magnitude of these errors are based on earlier DA studies (DeChant & Moradkhani, 2014; Yan et al., 2021). An ensemble size of 200 is used in this study to fully quantify the biomass and water temperature posteriors.

#### 2.5.4 Forecast evaluation metrics

In this study, forecasting skill is evaluated by both the deterministic metric root mean squared error (RMSE) and the probabilistic metric mean continuous ranked probability score (MCRPS) (Hersbach, 2000; Sun et al., 2019) as

(21) $$\text{RMSE }=\sqrt{\frac{\sum_{t=1}^{M}{\left[E\left({\hat{y}}_{t}\right)-y_{t}\right]}^{2}}{M}},$$

(22) $$\text{MCRPS }=\frac{\sum_{t=1}^{M}\int {\left[F\left({\hat{y}}_{t}\right)-H\left({\hat{y}}_{t}\geq y_{t}\right)\right]}^{2}d{\hat{y}}_{t}}{M},$$

where ${\hat{y}}_{t}$ and $y_{t}$ are the forecast and corresponding observed values, M is the forecast period, $F\left({\hat{y}}_{t}\right)$ is the forecast cumulative distribution function, and H(∙) is the heavy side function. RMSE has a range of [0, ∞) and measures deviation between observations and forecast ensemble mean values; an RMSE closer to 0 value suggests higher accuracy of a forecast. MCRPS also has a range of [0, ∞) but measures both forecast accuracy and the uncertainty spread; an MCRPS closer to 0 value suggests higher forecasting skill and generally smaller uncertainty.

### 2.6 Experiment design

A total of four forecasting experiments were performed in this study to fully illustrate the potential benefits of DA systems in short‐term biomass forecast by comparing the simulated biomass to the observed biomass growth in the raceway ponds. The raceway pond cultivation was carried out at Delhi, California. The cultures were grown at near 29 cm depth in BG‐11 (Andersen, 2005). Nitrogen and phosphate concentrations were monitored to ensure that the cultures remain under nutrient‐replete conditions. The pH was kept at near 7.0 (±0.2) by sparging CO_2_, controlled by a pH‐feedback system. There was no apparent biological contamination observed during the data collection period for growth simulation.

**Figure 2:**
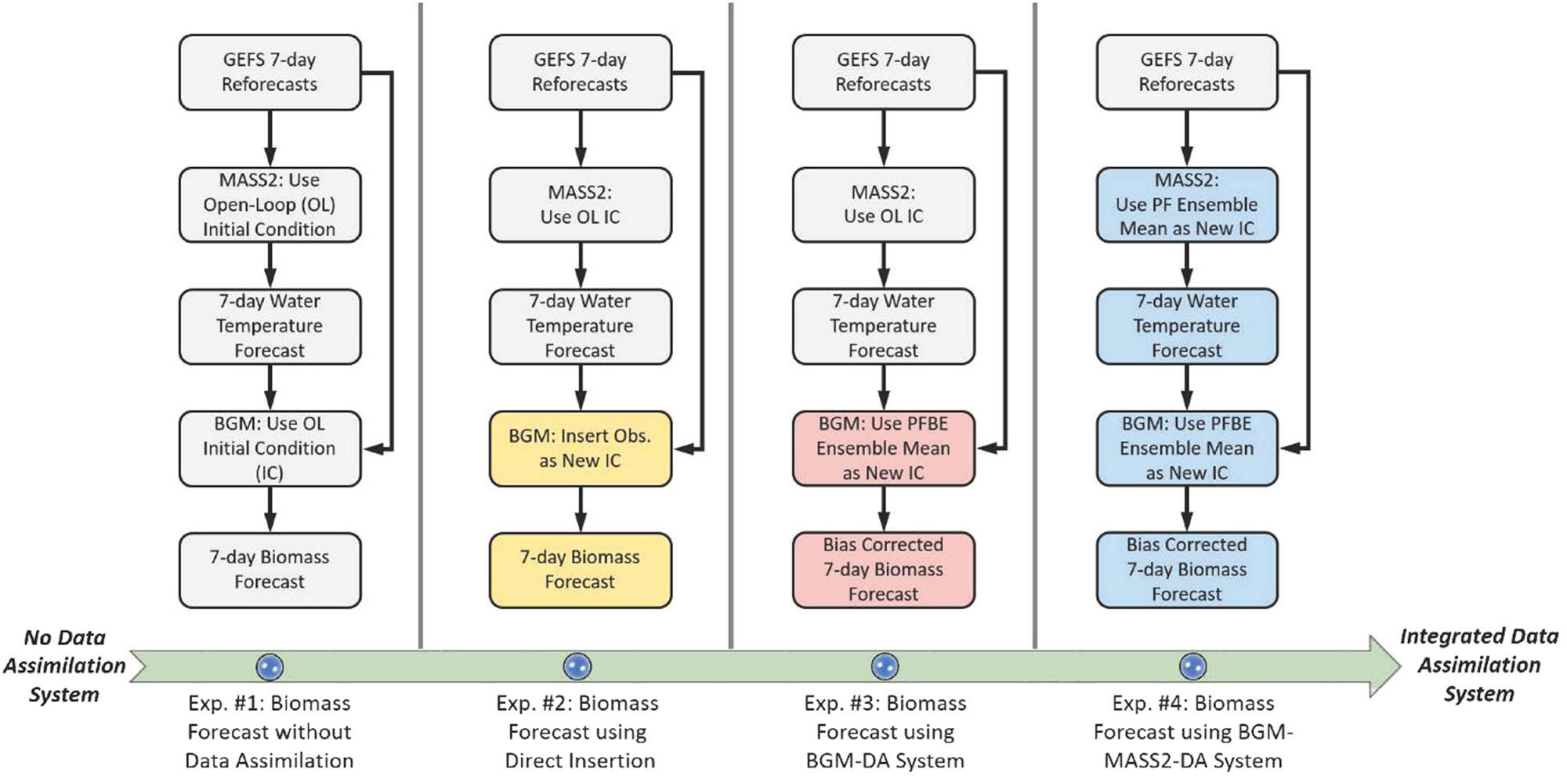
Illustration of four biomass forecasting experiments. Boxes with different colors show the differences among the experiments.

In Figure 2, it is observed the increasing sophistication of the forecasting systems from Exp. #1 to Exp. #4. Exp. #1 relied only on open‐loop (OL) simulations without ensemble DA as initial conditions for both MASS2 water temperature forecast and BGM biomass forecast. The forecasting skill from Exp. #1 is considered as a lower bound or benchmark in this study. Any improvements from the other three experiments come from the benefits of observations and the inclusion of ensemble DA systems. In Exp. #2, instead of relying on OL biomass concentration, we directly substituted BGM OL simulation by biomass observation as new initial conditions for forecasts. This rule‐based direct insertion (DI) method (Bernard et al., 1981) is straightforward to apply, but it does not consider observation uncertainty and BGM bias. Exp. #2 can also be considered as simply re‐running the BGM by manually re‐setting biomass initial conditions when new observations become available. Exp. #3 focused on the BGM‐DA system only (using MASS2 without DA) and used the PFBE method to quantify the biomass posteriors and selected the ensemble mean as new biomass initial conditions for forecasts. The ensemble mean of bias posteriors estimated at the forecasting initialization date is used to correct BGM biomass forecasts. Exp. #4 used the whole integrated BGM‐MASS2‐DA system. The only difference between the Exp. #3 and Exp. #4 is the inclusion of the MASS2‐DA system to enhance the water temperature forecast. We used the PF method to update MASS2 water temperature simulations and selected ensemble mean as new MASS2 initial conditions for water temperature forecasts. In all four experiments, we iteratively generated near‐term (i.e., 7‐day) biomass forecasts once new biomass measurements (i.e., OD_750_) become available.

## 3 RESULTS AND DISCUSSION

In the following, the 7‐day biomass forecasting results of Exp. #1 to Exp. #4 are reported. Both OL and DA spin‐up simulations (i.e., no forecasting) of MASS2 and BGM are first presented in Section 3.1. We compare OL and DA results to assess the potential benefits of using DA to improve water temperature and biomass simulations, and consequently, the initial conditions. Second, Exp. #1 OL biomass forecasting results and Exp. #2 DI biomass forecasting results are presented in Section 3.2. Exp. #3 BGM‐DA biomass forecasting results and Exp. #4 integrated BGM‐MASS2‐DA biomass forecasting results are presented in the following Section 3.3. In this section, we also compare MASS2 OL and DA water temperature forecasting results and discuss the pros and cons of each forecasting system.

### 3.1 MASS2 and BGM OL and DA spin‐up simulations

**Figure 3:**
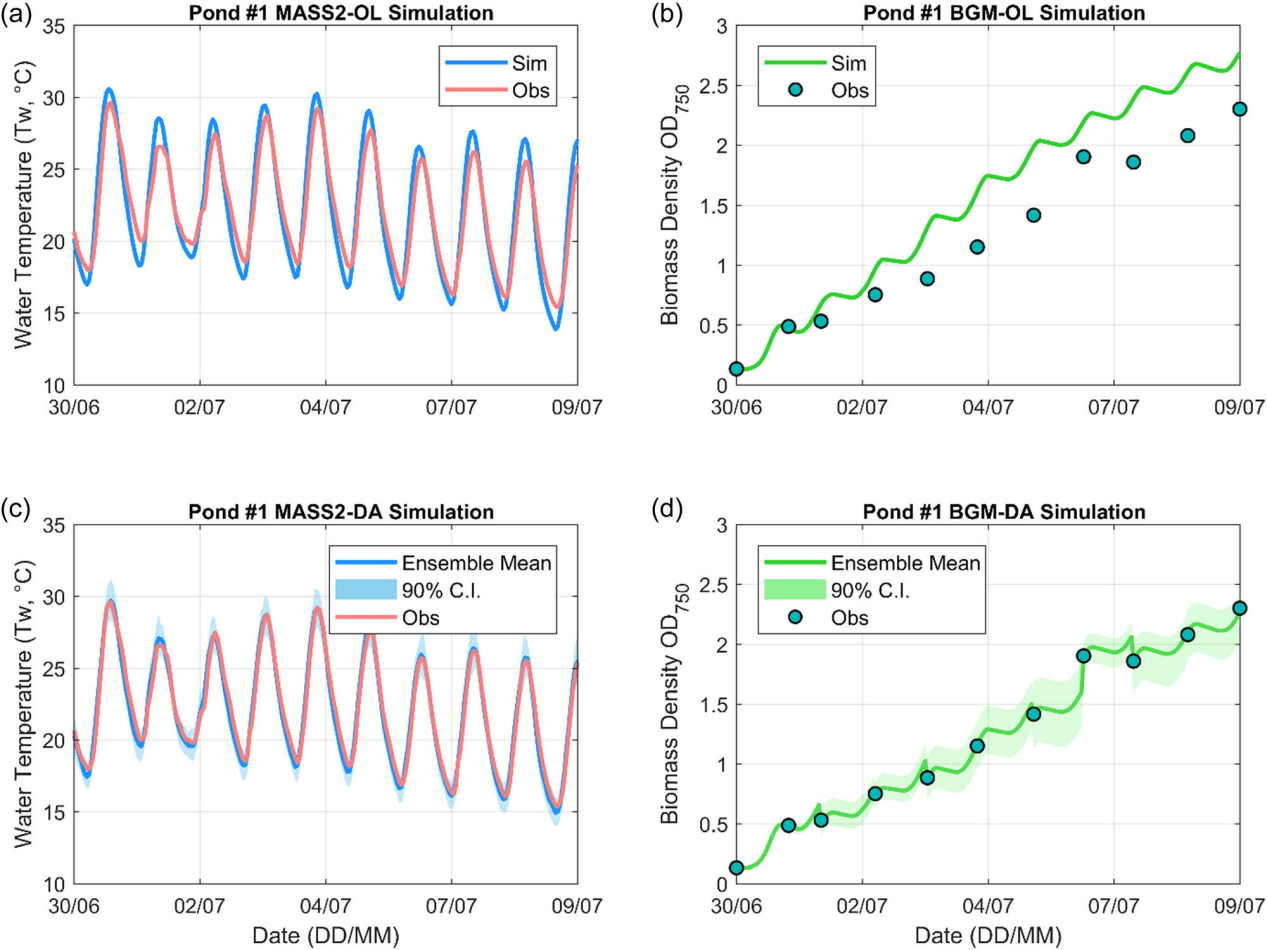
Open‐loop (OL) and data assimilation (DA) MASS2 and BGM simulations for Pond #1

MASS2 water temperature and BGM biomass simulations for pond #1 are both shown in Figure 3. Note that MASS2 was driven by local CIMIS meteorological forcing data while BGM was driven by on‐site measured pond water temperature and PAR. It is observed that MASS2 OL simulations moderately overestimated water temperature at daytime and underestimated water temperature at nighttime while DA simulations helped to correct these biases. For the biomass case, despite using more reliable forcing data (i.e., on‐site measurements), BGM OL results still showed systematic overestimation against biomass observations. These results are not surprising and have been reported in our previous studies (Gao et al., 2018; Huesemann et al., 2016). The realistic nonoptimal outdoor growth conditions and biomass parameter uncertainties together contribute to the biased simulations and limit the potential BGM operational implementation to guide algae operation and harvesting (Yan et al., 2021). The standard PF algorithm cannot address the BGM systematic overestimates while the proposed PFBE can quantify the bias uncertainty. The ensemble biomass simulation results shown in Figure 3d were bias‐corrected. Similar results were observed for the other two ponds (Supporting Information: Figures S1 and S2). The deterministic RMSE values for both OL and DA simulations are summarized in Table 1 and indicate that the DA simulations substantially outperform the corresponding OL simulations. For instance, the RMSE of water temperature decreases from 1.25°C for the OL simulation to 0.48°C for the DA simulation, indicating a 62% improvement in model prediction. The RMSE of biomass density in terms of OD_750_ decreases from 0.39 for the OL simulation to 0.02 for the DA simulation (i.e., 95% improvement), suggesting a more accurate characterization of initial conditions for biomass forecasting.

**Table 1:** Root mean squared error (RMSE) of MASS2 and BGM open‐loop (OL) and data assimilation (DA) simulations for Pond #1

Simulation	Variable	RMSE
MASS2‐OL	Water temperature (°C)	1.25
MASS2‐DA Ensemble mean	Water temperature (°C)	0.48
BGM‐OL	Biomass density (OD_750_)	0.39
BGM‐DA Ensemble mean	Biomass density (OD_750_)	0.02

### 3.2 Biomass forecasts without ensemble DA

In the following, we report forecasting results from Exp. #1 and Exp. #2 that are results without using the ensemble DA method. Specifically, Exp. #1 used biased OL simulation results (see Figure 3b) as biomass initial conditions while Exp. #2 directly replaced OL simulation results with biomass measurements to generate forecasts. Both Exp. #1 and Exp. #2 used the same operational hydrometeorological forecasts: PAR forecasts were directly taken from GEFS and water temperature forecasts were provided by MASS2 driven by GEFS using OL initial condition (see Figure 3a).

#### 3.2.1 Exp. #1: Open‐loop biomass forecasts

**Figure 4:**
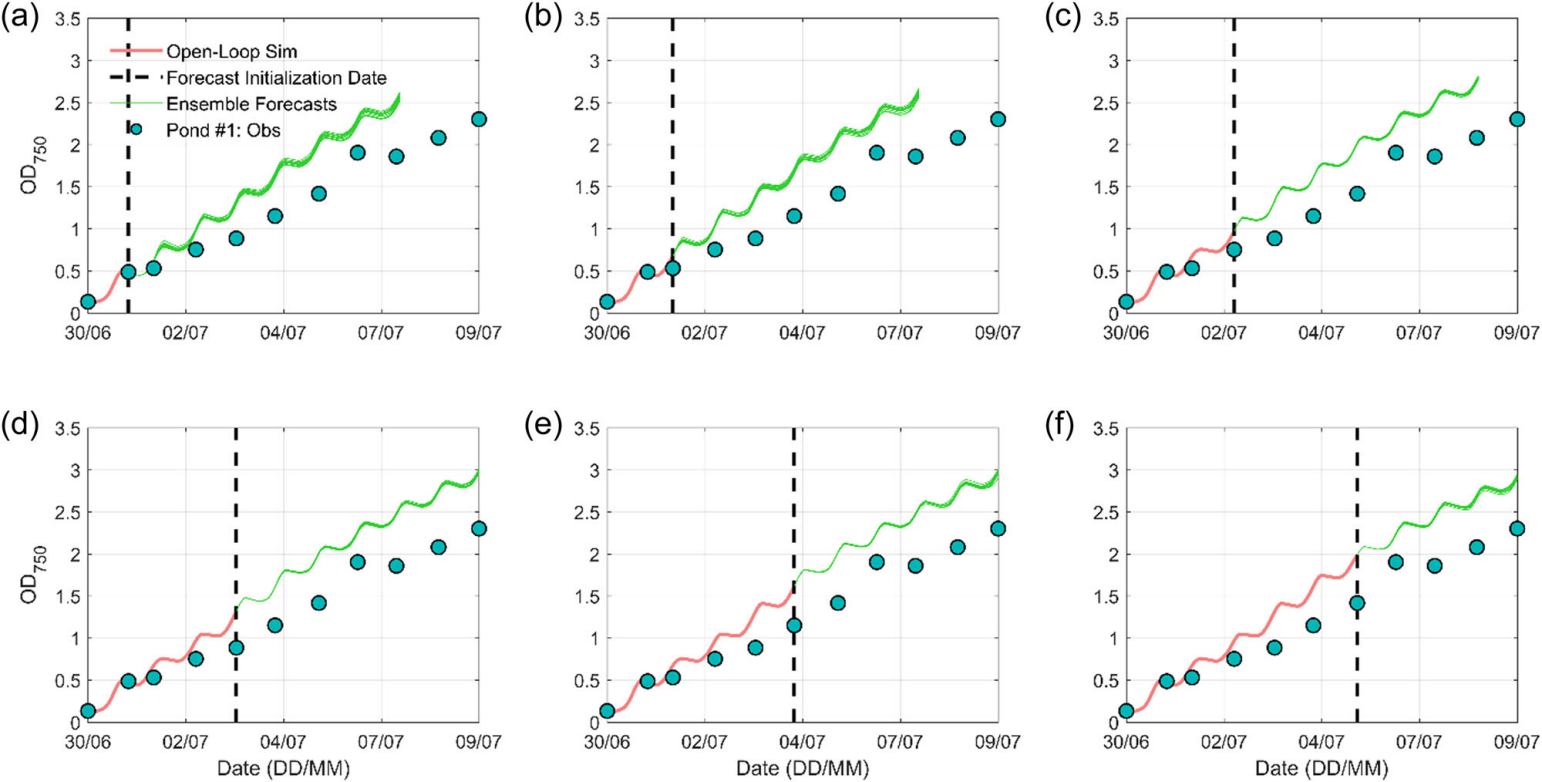
Six open‐loop (OL) 7‐day microalgae growth forecasts for Pond #1. The black dashed line indicates each forecasting initialization date when a new observation became available. The red curve indicates the BGM OL simulation using observed light and water temperature (i.e., spin‐up period). The green curves indicate BGM 11 ensemble forecasts using the OL simulation as initial condition. Note in (d, e, f), only 6‐, 5‐, and 4‐day forecasts are presented to match the available biomass observations.

Figure 4 presents six 7‐day ensemble biomass forecasts initialized when new biomass measurements became available for Pond #1. All six forecasts using biased OL initial conditions generated substantially systematic overestimations. These results are expected and consistent with Yan et al. (2021) who quantified the relative importance of initial condition and meteorological forecast skill to the 7‐day biomass forecast and found out that accurate characterization of initial condition played a more prominent role. Similar performances were observed for the other two ponds (Figures S3 and S4). Both deterministic RMSE values of the ensemble mean forecast and probabilistic MCRPS values of 11 ensemble forecasts in Figure 4 are summarized in Table 2. For each forecast, we report the average 7‐day forecasting skill instead of the daily forecasting skill because biomass density was not measured at a consistent time interval. These OL biomass forecasting results were the benchmark or lower bound and any improvement achieved from the other three experiments can be considered the added benefits of biomass or water temperature measurements.

**Table 2:** Performances of short‐term biomass forecasts (in terms of OD_750_) for the four experiments in Pond #1

Metric	experiment	Forecast number/subplot number						
		(a)	(b)	(c)	(d)	(e)	(f)	Avg.
RMSE	Exp. #1: OL Forecast	0.45	0.53	0.54	0.57	0.59	0.55	0.54
	Exp. #2: DI Forecast	0.43	0.34	0.24	0.11	0.11	0.13	0.23
	Exp. #3: BGM‐DA Forecast	0.41	0.29	0.18	0.10	0.10	0.16	0.21
	Exp. #4: BGM‐MASS2‐DA Forecast	0.41	0.29	0.18	0.10	0.10	0.16	0.21
MCRPS	Exp. #1: OL Forecast	0.40	0.50	0.52	0.56	0.58	0.53	0.51
	Exp. #2: DI Forecast	0.39	0.30	0.21	0.09	0.10	0.08	0.20
	Exp. #3: BGM‐DA Forecast	0.38	0.26	0.16	0.06	0.07	0.14	0.18
	Exp. #4: BGM‐MASS2‐DA Forecast	0.38	0.26	0.16	0.06	0.07	0.13	0.18

Abbreviations: MCRPS, mean continuous ranked probability score; RMSE, root mean squared error.

#### 3.2.2 Exp. #2: Direct insertion biomass forecasts

**Figure 5:**
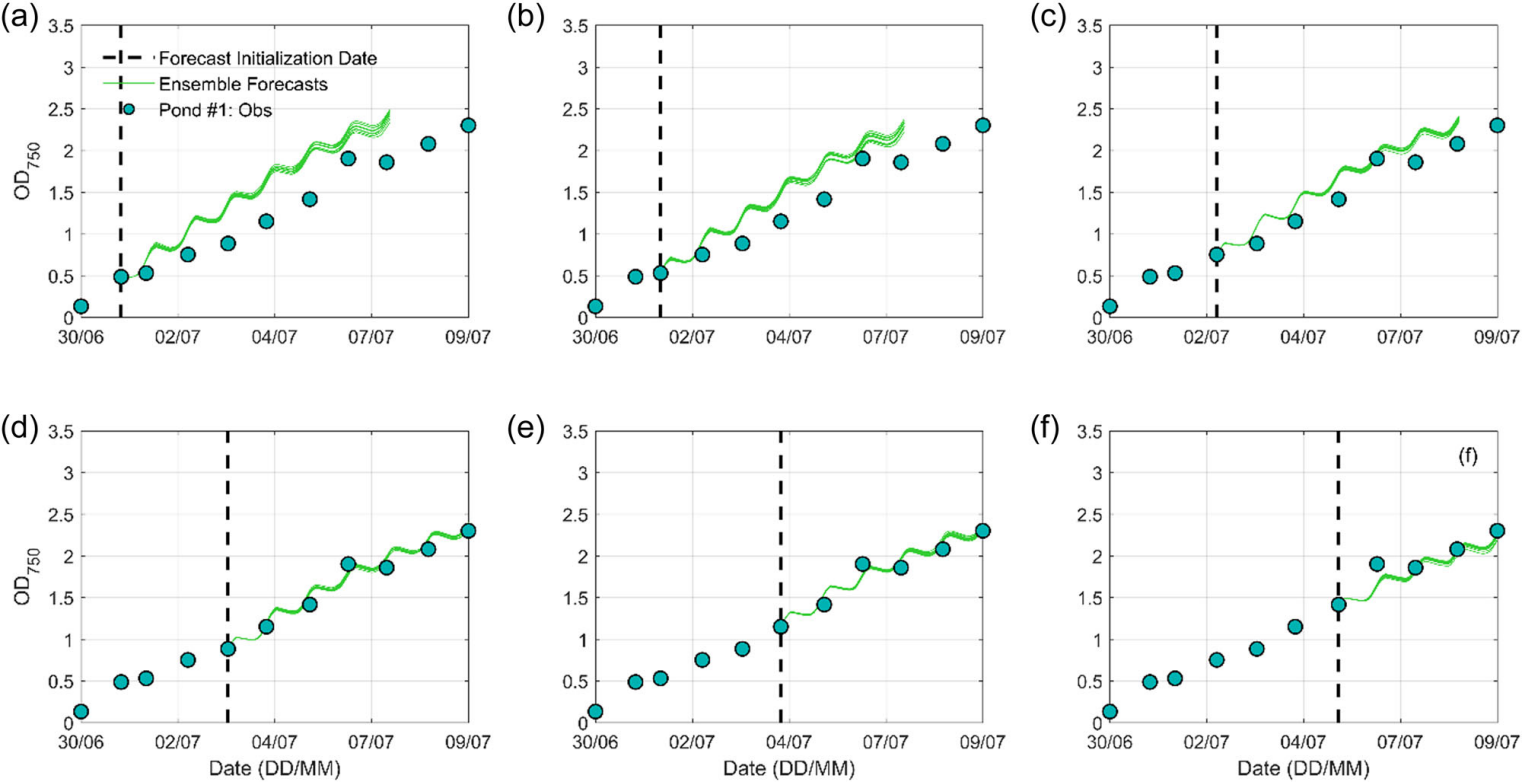
Six direct insertion (DI) 7‐day microalgae growth forecasts for Pond #1. The black dashed line indicates each forecasting initialization date when a new observation became available. The green curves indicate BGM 11 ensemble forecasts using the observations as initial conditions. Note in (d–f), only 6‐, 5‐, and 4‐day forecasts are presented to match the available biomass observations.

Figure 5 presents six 7‐day ensemble biomass forecasts for Pond #1 using the DI method and Table 2 summarizes the RMSE and MCRPS values. Compared to the OL forecasts, the DI biomass forecasts showed higher forecasting skills. For example, the RMSE and MCRPS values for the OL biomass forecast initialized on July 2, 2015 were 0.54 and 0.52, and they decreased to 0.24 and 0.21 in the DI biomass forecast, respectively. Figures 5d–f also suggested acceptable 7‐day forecasting results in this study. Similar results were found for the other two ponds (Supporting Information: Figures S5 and S6). These results suggest biomass short‐term forecasts can be improved by improving initial conditions by directly inserting biomass measurements. Despite the use of the DI method, after the initialization date, the BGM forecasts may quickly drift back to a biased state (e.g., Figure 5c) due to the nonoptimal outdoor growth conditions and model parameter uncertainties which also depend on the local climate variability (Sun et al., 2019). When and how the model bias affects the DI forecasting results are unpredictable. As a result, these potentially biased DI forecasts are unreliable and insufficient to inform pond operation and harvesting planning.

### 3.3 Integrated BGM‐MASS2‐DA biomass forecasts

In this section, we present forecasting results from Exp. #3 and Exp. #4 that are results from using ensemble DA. Both experiments used the PFBE method to assimilate biomass measurements to improve BGM's initial conditions and quantify model biases. Same as Exp. #1 and Exp. #2, Exp. #3 used the water temperature forecasts that were provided by MASS2 driven by GEFS using the OL initial condition. While Exp. #4 used the PF method to update MASS2 initial condition by assimilating water temperature measurements and generated the 7‐day water temperature forecasts using the updated DA initial condition.

#### 3.3.1 Exp. #3: BGM‐DA biomass forecasts

**Figure 6:**
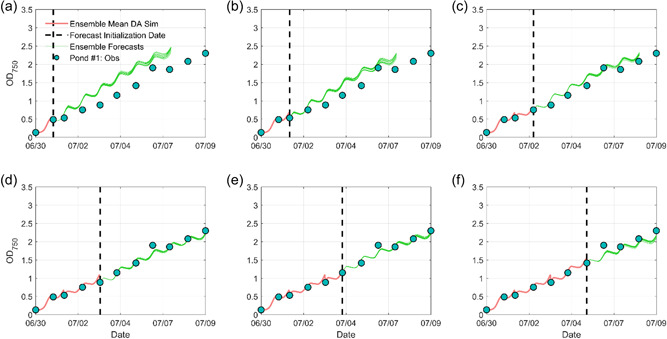
Six BGM‐DA 7‐day microalgae growth forecasts for Pond #1. The black dashed line indicates each forecasting initialization date when a new observation became available. The red curve indicates the BGM‐DA simulation before the forecast initialization date. The green curves indicate BGM 11 ensemble forecasts using the observations as initial conditions. Note in (d–f), only 6‐, 5‐, and 4‐day forecasts are presented to match the available biomass observations.

Figure 6 presents the six ensemble biomass forecasts with the assimilation of biomass measurements. Compared to the forecasts using the DI method, the overestimations were substantially reduced after the early culture adaption stage. The performance metrics were summarized in Table 2. For example, in Figure 6c, the RMSE value decreased from 0.24 in the DI forecast to 0.18 in the BGM‐DA forecast and the MCRPS value decreased from 0.21 in the DI forecast to 0.16 in the BGM‐DA forecast, respectively. Compared to Exp. #1, it is also observed that the improvement rates of using the BGM‐DA method were smaller in the early growth stage. From a technical perspective, this is due to the relatively coarse temporal resolution of biomass measurements (i.e., daily time step). Model bias evolved quickly in the exponential growth stage and the bias estimated in the early stage could not represent the bias in the later exponential growth stage. From a biological perspective, this is associated with incomplete acclimation of the culture or imperfect characterization of the organism at low light and temperature ranges (Yan et al., 2021). As the forecast date advances with more measurements (e.g., Figures 6d–f), the BGM‐DA system can better capture the model bias because there is more information from previous periods, leading to better biomass forecasts. Nevertheless, even in the early growth stage, the BGM‐DA system still showed higher forecasting skills than the OL and DI system. For example, in Figure 6b, the RMSE value decreased from 0.34 in the DI forecast to 0.29 in the BGM‐DA forecast. Similarly, we also found that BGM‐DA forecasts were superior to DI forecasts for the other two ponds (Supporting Information: Figures S7 and S8). These results demonstrate the importance of bias quantification in biomass forecasts.

#### 3.3.2 Exp. #4‐1: MASS2‐DA water temperature forecasts

**Figure 7:**
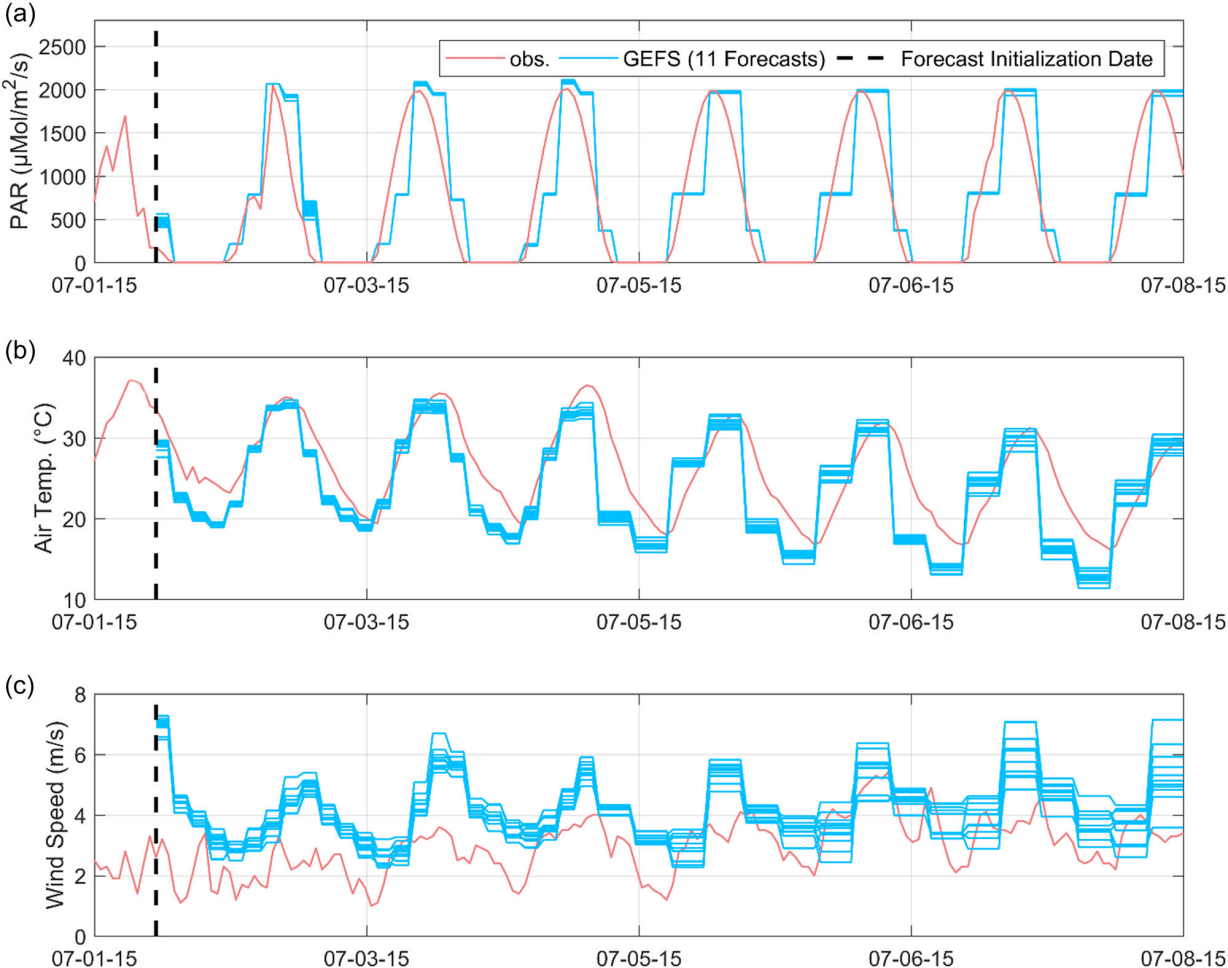
One GEFS 7‐day meteorological forecasts versus the CIMIS observation data. Note the biased air temperature and wind speed forecasts, leading to biased water temperature forecasts using MASS2.

**Figure 8:**
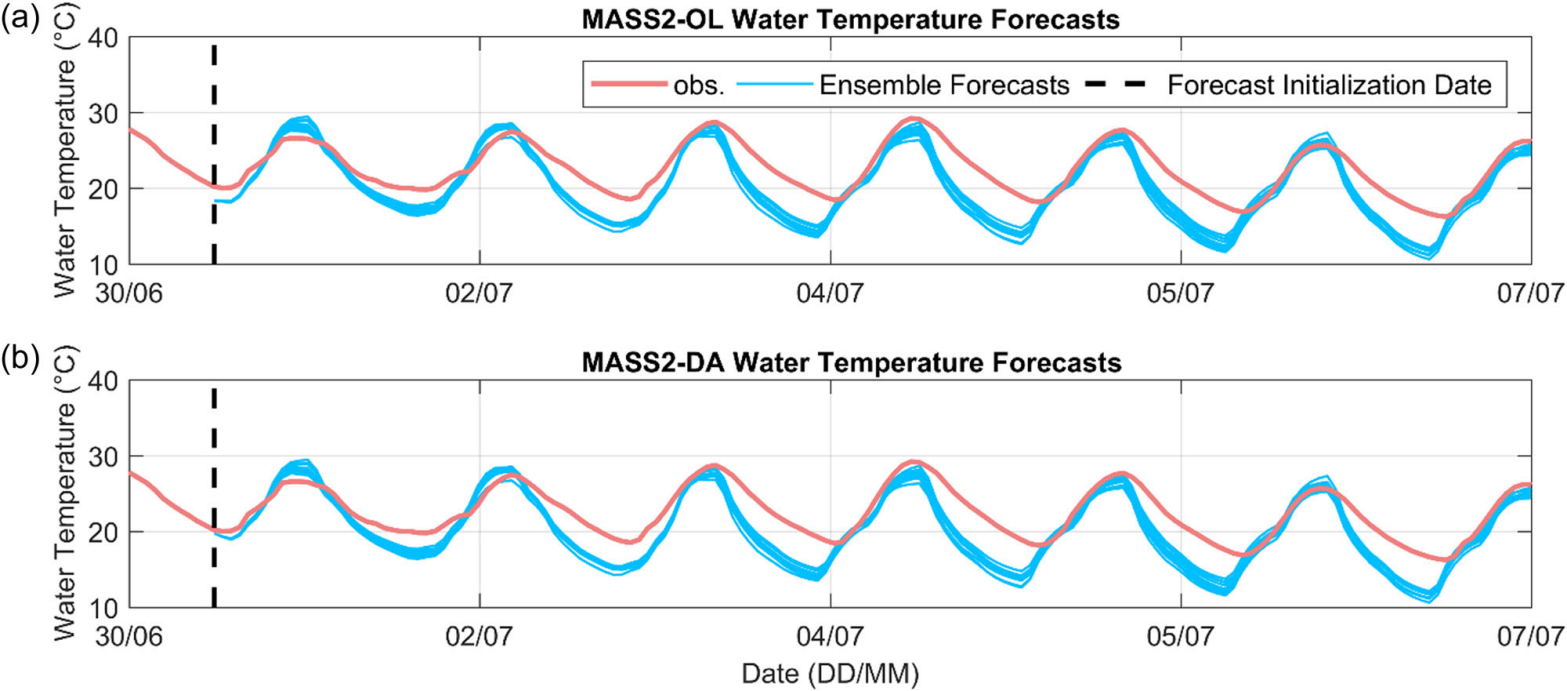
(a) One MASS2‐OL 7‐day water temperature ensemble forecast using OL simulation as the initial condition at 2015‐07‐01: 17:00 for Pond #1. (b) One MASS2‐DA 7‐day water temperature forecast using DA updated initial condition at the same date and time for Pond #1.

All of the above forecasting results rely on the MASS2‐OL water temperature forecasts without the assimilation of water temperature data. Pond water temperature data were automatically measured at a finer time step (i.e., hourly) and can be used to improve MASS2 initial conditions for the water temperature forecast. Based on the available pond temperature measurements, we generated 13 MASS2‐OL and MASS2‐DA 7‐day water temperature forecasts initialized at 17:00 from 30 June, 2015 to 12 July, 2015. Figure 7 presents one example of the GEFS ensemble meteorological forecasts versus the CIMIS observation data, suggesting the underestimation of air temperature and the overestimation of wind speed. Figure 8 shows one MASS2‐OL and one MASS2‐DA forecast as examples. In Figure 8, it is observed that the water temperature initial condition on the forecasting initialization date was improved by assimilating water temperature data, leading to improved water temperature forecasts in the earlier leading time. On the 7‐day scale, we found that MASS2‐DA only slightly improved MASS‐OL forecasting skill for all 13 forecasts for this specific site (e.g., the average RMSE value decreased from 2.935°C in the MASS2 OL forecast to 2.927°C in the MASS2 DA forecast), suggesting the less dominant role of the initial condition on water temperature forecasts. In this case, the skills of meteorological forecasts also play an important role; quantifying the relative roles of initial conditions and meteorological forecasts in pond water temperature need further study. In summary, we expect the impacts of the MASS‐DA forecast to depend on the local climate, GEFS forecast skill, and pond conditions (e.g., above‐ground pond vs. inground pond and operating depth). Our preliminary results in another study suggested larger improvements using MASS2‐DA for inground ponds than above‐ground ponds.

#### 3.3.3 Exp. #4‐2: BGM‐MASS2‐DA biomass forecasts

**Figure 9:**
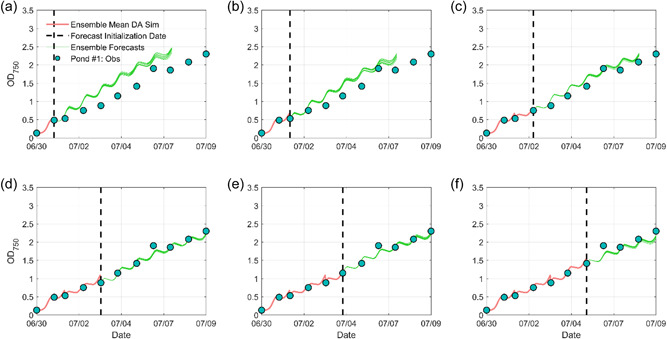
Six integrated BGM‐MASS2‐DA 7‐day microalgae growth forecasts for Pond #1. The black dashed line indicates each forecasting initialization date when a new observation became available. The red curve indicates the BGM‐MASS2‐DA simulation before the forecast initialization date. The green curves indicate BGM 11 ensemble forecasts using the observations as initial conditions. Note in (d–f), only 6‐, 5‐, and 4‐day forecasts are presented to match the available biomass observations.

Figure 9 presents the integrated BGM‐MASS2‐DA biomass forecasts for Pond #1 and these performance metrics are summarized in Table 2. On average of the six forecasts, the integrated system improved the OL, DI, and BGM‐DA forecasts by about 62%, 8%, 0.23% in RMSE value and 66%, 11%, and 0.30% in MCRPS value, respectively. The minor improvement between BGM‐DA and BGM‐MASS2‐DA is due to the moderate improvements in the water temperature forecasts as discussed above. Despite only minor improvements, it is still better to use the measured water temperature data and update the MASS2 initial condition to generate better water temperature forecasts, because the results found for this specific location (i.e., Delhi, California) may not be the same for other locations with different GEFS forecasting skill, climate, and pond operating conditions. Similar performances were also observed for the other two ponds (Supporting Information: Figures S9 and S10). Given the satisfactory performances achieved in this study, it is probable that the integrated BGM‐MASS2‐DA forecasting system can be used to guide pond operation and harvesting planning. For example, consider the circumstance in which a manager plans to harvest biomass in 3 days, but the latest forecast suggests the biomass growth rate will decrease after 2 days because the weather will get colder. To achieve maximum biomass productivity, the manager will schedule harvesting for 2 days. Or if the manager plans to harvest 500 kg biomass in 3 days, but the latest forecast suggests that there will be only a 35% probability of having 500 kg biomass in 3 days and about 90% probability of having 500 kg biomass in 7 days. From an economic point of view, the manager will reschedule the harvesting planning resources to 7 days. Or if the forecast suggests that the biomass density will be 1.4 g L^−1^ in 3 days, but the biomass measurement after 3 days is only 0.7 g L^−1^ despite the weather forecast being accurate, suggesting an “abnormal” condition (e.g., biological contamination or nutrient deficiency) in the pond and alarming immediate pond operation such as harvesting to avoid losing the existed biomass.

In this study, we only generate a 7‐day biomass forecast despite the GEFS providing up to 15‐day weather forecasts. We provide a 7‐day biomass forecast mainly for two reasons: (1) The 7‐day weather forecast is more reliable and larger error increases on the later days. For example, the forecast accuracy for the coming seventh day is about 75% while for the 10th day is only about 45% (Bauer et al., 2015). Large errors in weather forecasts after 7 days will propagate into water temperature and biomass forecasts, leading to less reliable biomass forecasts to guide pond operation. (2) A 7‐day forecast should be sufficient enough for pond operation decisions such as optimizing dilution rate (Gao et al., 2022). GEFS weather forecast is updated each day and we iteratively update a 7‐day biomass forecast each day. In practice, most of the time a 3‐day forecast is sufficient enough for planning purposes such as determining pond dilution rate. In addition, besides the short‐term 7‐day biomass forecast as validated in this paper, another application of this forecasting application is to use the seasonal climate predictions such as the North American Multi‐Model Ensemble (Becker et al., 2014) to generate long‐term (monthly to seasonal) biomass predictions to guide long‐term pond operation decision such as strain rotation strategy (e.g., which strain is anticipated to have the highest productivity in the coming summer season). Finally, this integrated biomass forecasting system is flexible to adopt another type of microalgae rather than the *Chlorella sorokiniana*. All the users need to do is changing the strain parameters (i.e., growth parameters as a function of both temperature and light intensity) inside the BGM. One previous example is the use of the *Monoraphidium minutum* 26B‐AM by Yan et al. (2021).

## 4 CONCLUSIONS

In this study, we proposed an integrated BGM‐MASS2‐DA biomass forecasting system consisting of the BGM biomass model, MASS2 water temperature model, ensemble DA, and NWP GEFS meteorological forecasts. The integrated forecasting system was implemented in three outdoor ponds cultured with *Chlorella sorokiniana* in Delhi, California, United States. A total of four experiments were designed, with the increasing sophistication of the forecasting system, to fully quantify the value of the integrated system. We examined the impacts of assimilating water temperature and biomass measurements for quantifying the initial conditions and their subsequent contributions toward improved forecasting of water temperature and biomass. Results from three ponds suggest that the proposed integrated biomass forecasting system improved the short‐term (i.e., 7‐day) biomass forecasting skill by about 60% on average compared to forecasts without DA. The satisfactory performances achieved in this study suggest the potential of using the integrated forecasting system to facilitate pond operations and harvesting planning such as optimizing pond dilution rate.

Last, we acknowledge that further improvements can be made to enhance the integrated BGM‐MASS2‐DA forecasting system. Specifically, we outline the following five directions. (1) Improve BGM by adding a nutrient component. Because the BGM was built based on nutrient‐replete axenic laboratory cultures, the proposed method can only be applied to cultures where nutrients are not limiting. Incorporation of a nutrient component into the BGM could further improve the applicability of the proposed method for cases such as enhancing lipid accumulation by nitrogen depletion. (2) Update BGM parameters in the DA system. Dual state‐parameter DA updating (i.e., updating the model states and parameters at the same time) is not new and has been studied for years (Abbaszadeh et al., 2018; Yan et al., 2015). In this study, we did not adjust *Chlorella sorokiniana* growth parameters but treated the parameter uncertainty in the bias term, because algal growth parameters were determined experimentally as a function of both temperature and light intensity in laboratory cultures. Due to cost, it is infeasible to generate parameters in the laboratory under all circumstances. One possible solution to use dual state‐parameter DA updating is to carefully predefine a range/uncertainty for algal growth parameters using both experimental values and expert judgment. (3) Generate continuous biomass observations (OD_750_). The biomass density in this study was manually measured daily or once in 2 days. The coarse measurement resolution resulted in suboptimal DA performances and limited biomass forecasting skills in the early growth stage or when a pond is under frequent harvesting or changing operating conditions (e.g., dilution). We plan to use automatic OD_750_ sensors to provide continuous, fine temporal resolution of biomass density measurements. In such a case, biomass forecasts can be generated at a fine time step (e.g., hourly) and better guide pond operation and harvesting. (4) Test the forecasting system in semicontinuous operations for long periods (i.e., months). We understand that industrial culture facilities are generally designed to operate continuously for several months in semicontinuous mode. Harvesting interferes in semicontinuous mode affect the accumulation of metabolic products and stress the culture (e.g., less mixing, sudden increase in light penetration and nutrients) and the BGM does not accommodate these effects using the calibrated parameters, which further suggests the importance of quantifying parameter uncertainty especially in the early growth stage. We are especially interested in examining whether the forecasting system can guide operation by optimizing the harvesting time and dilution rate to maximize biomass production. Our preliminary field study suggested that the weather‐forecast‐informed dilution strategy improved the biomass productivity by 47% over the standard batch cultivation and 20% over the fixed‐rate dilution case (Gao et al., 2022). (5) Investigate the MASS2 water temperature forecasting skills. This study found that after a few time steps, the MASS2‐DA did not improve MASS2‐OL water temperature forecasts. The relative role of the initial condition and meteorological forecasting skills at different locations on biomass forecasts under different pond operating conditions needs further studies.

## AUTHOR‐CONTRIBUTIONS

**Hongxiang Yan**: Conceptualization; data acquisition; data analysis; methodology; writing – original draft. **Mark S. Wigmosta**: Funding acquisition; methodology; data acquisition; writing – review & editing. **Michael H. Huesemann:** Methodology; data acquisition; writing – review & editing. **Ning Sun**: Methodology; data acquisition; writing – review & editing. **Song Gao**: Methodology; data acquisition; writing – review & editing.

## CONFLICT OF INTEREST

The authors declare no conflict of interest.

## Supporting information

Supplementary information.

## Data Availability

The local meteorological data at Delhi, California were acquired at: https://cimis.water.ca.gov/. The GEFS forecasts data were acquired at: https://psl.noaa.gov/forecasts/reforecast2/download.html The data that support the findings of this study are available from the corresponding author upon reasonable request.
