# Supplementary material for: An ensemble data assimilation modeling system for operational outdoor microalgae growth forecasting
Source: Biotechnol Bioeng. 2022 Nov 6;120(2):426–43. doi: 10.1002/bit.28272 (PMC10098620; doi:10.1002/bit.28272)
Supplement: Supplementary file 1 — Supplementary information. [file BIT-120-426-s001.docx]

**Supporting Information (SI)**

**An Ensemble Data Assimilation Modeling System for Operational Outdoor Microalgae Growth Forecasting**

Hongxiang Yan^1*^, Mark S. Wigmosta^1,2^, Michael H. Huesemann^3^, Ning Sun^1^, and Song Gao^3^

^1^ Energy and Environment Directorate, Pacific Northwest National Laboratory, Richland, Washington 99352, United States

^2^ Department of Civil and Environmental Engineering, University of Washington, Seattle, Washington 98195, United States

^3^ Marine and Coastal Research Laboratory, Pacific Northwest National Laboratory, Sequim, Washington 98382, United States

*Corresponding author.

Hongxiang Yan: hongxiang.yan@pnnl.gov

**
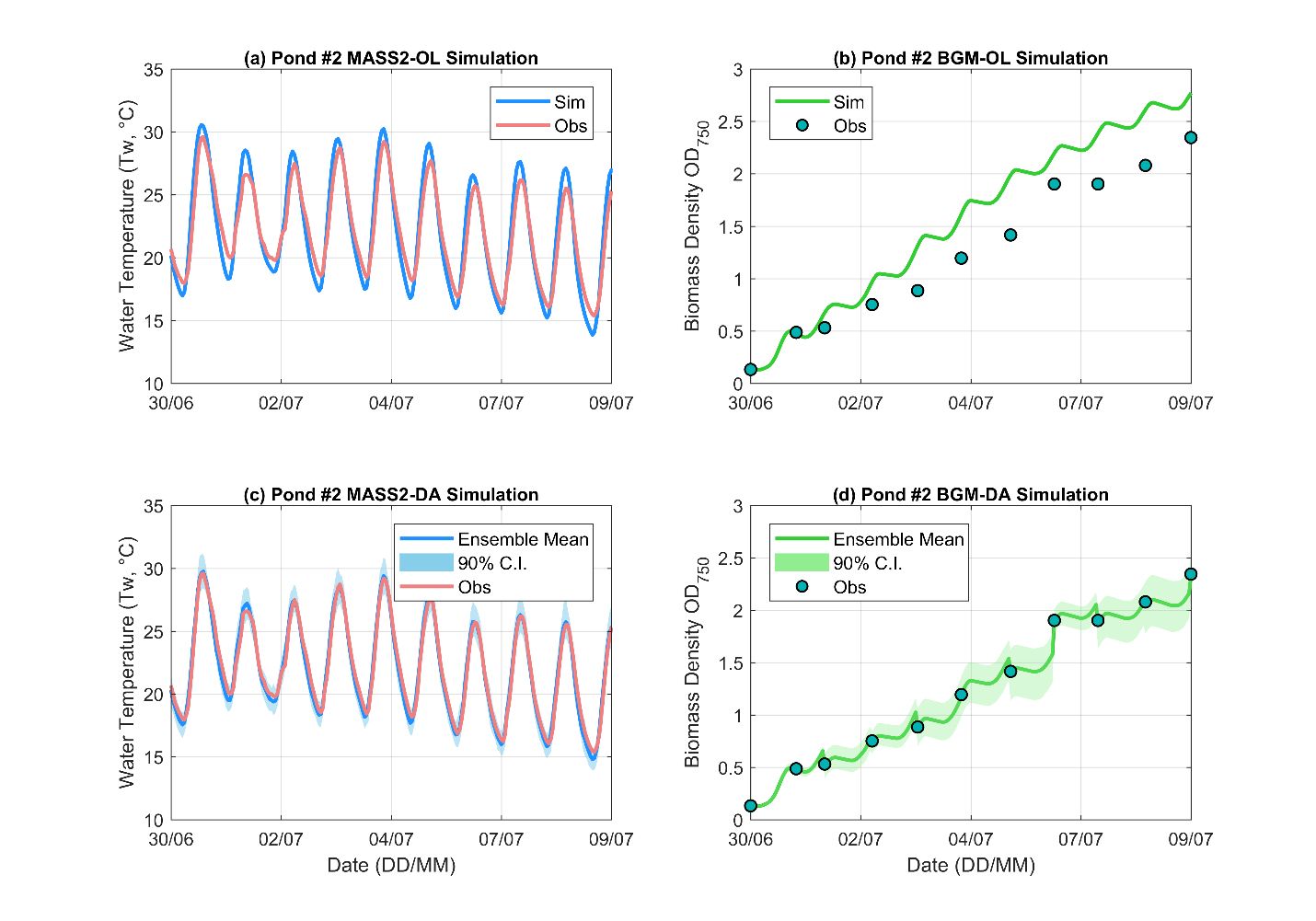
**

**Figure S1.** Open-loop (OL) and data assimilation (DA) MASS2 and BGM simulations for pond #2.


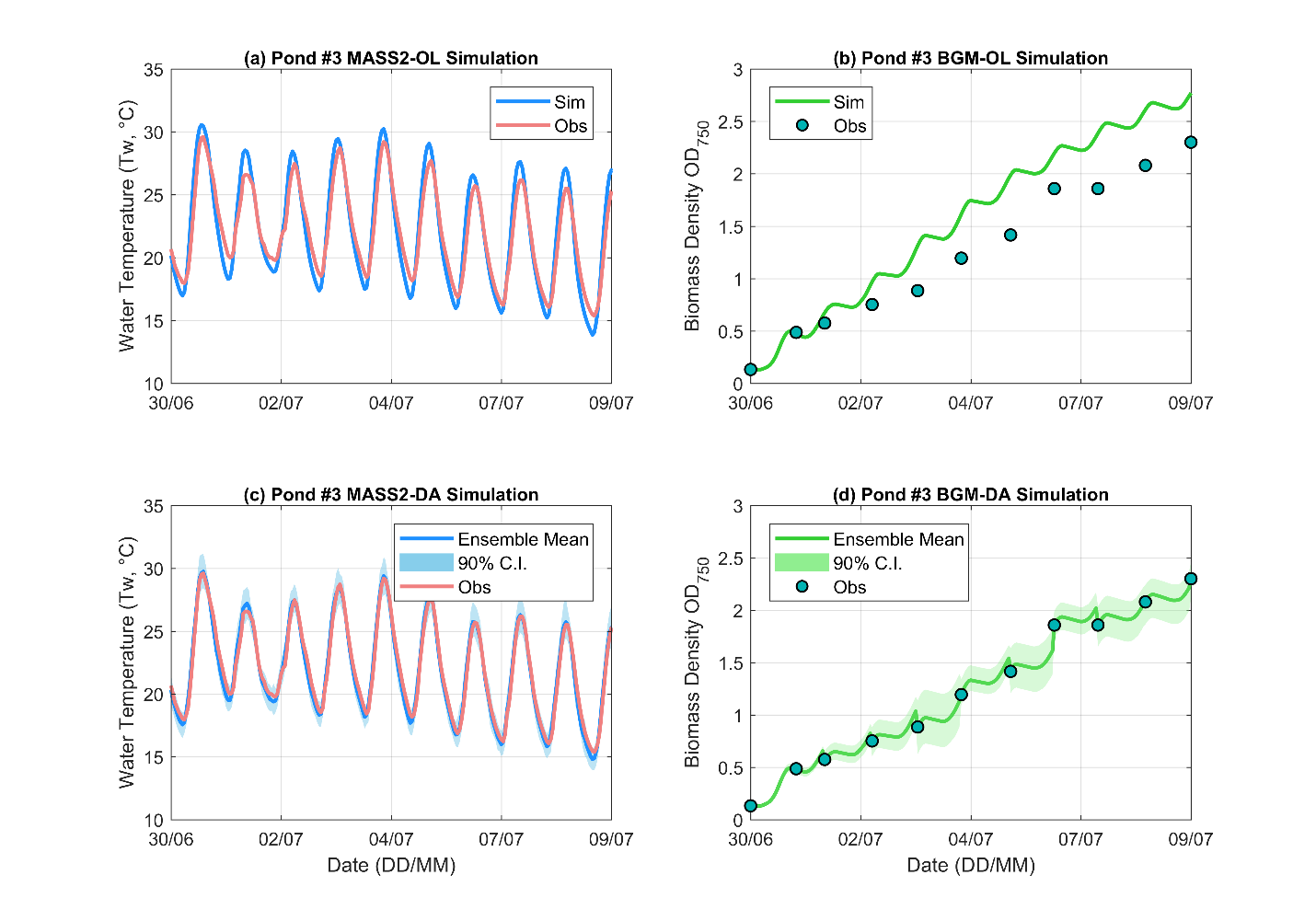


**Figure S2.** Open-loop (OL) and data assimilation (DA) MASS2 and BGM simulations for pond #3.


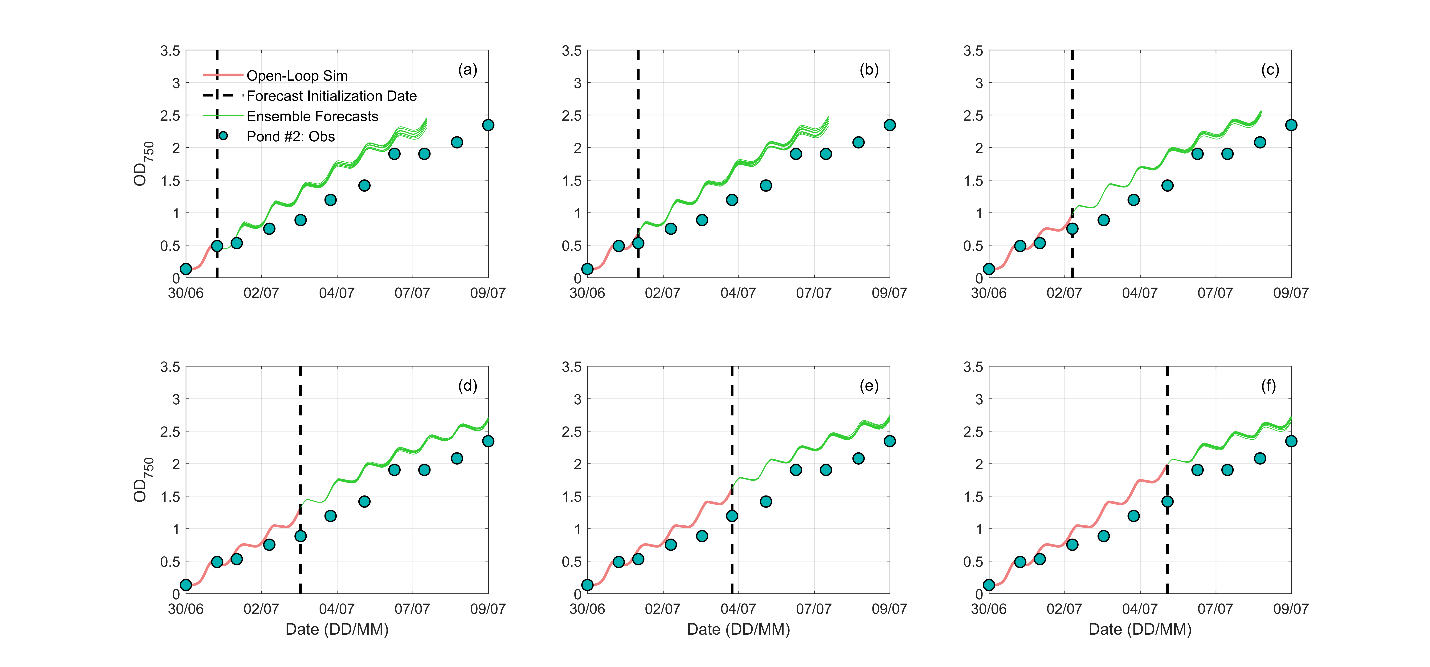


**Figure S3.** Six open-loop (OL) 7-day microalgae growth forecasts for pond #2.


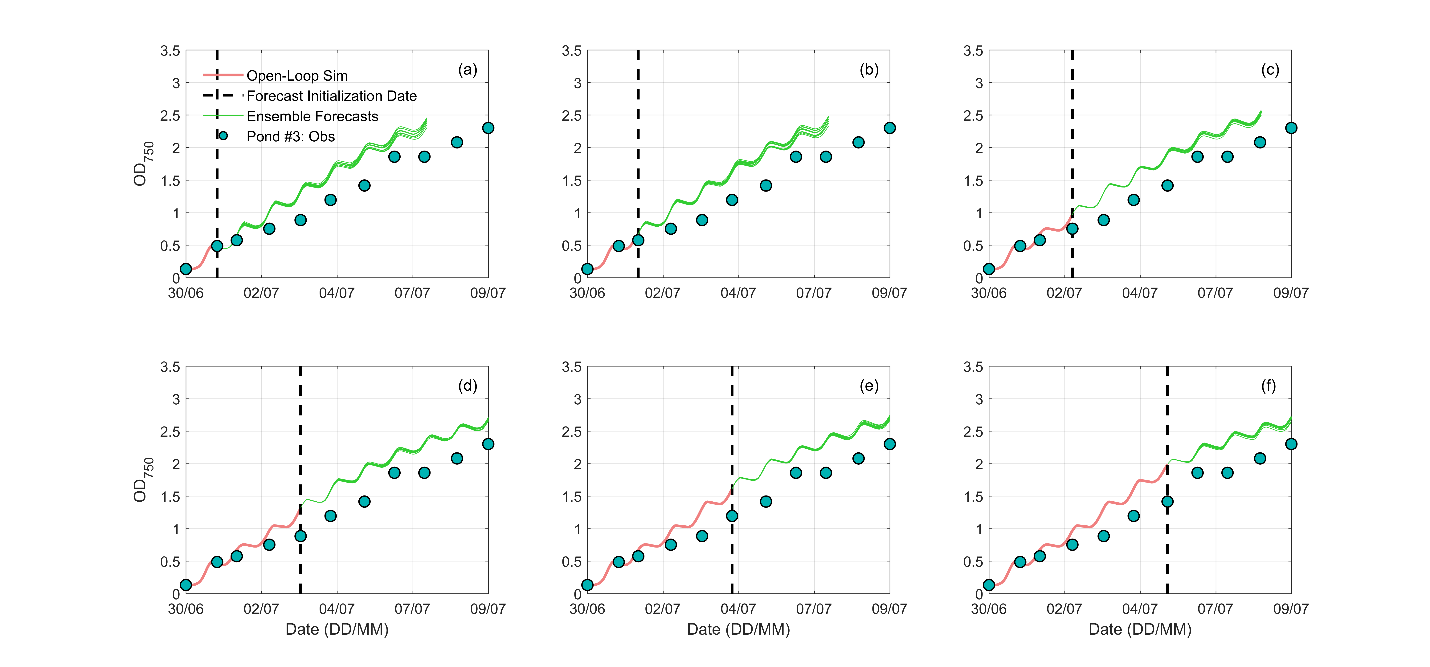


**Figure S4.** Six open-loop (OL) 7-day microalgae growth forecasts for pond #3.


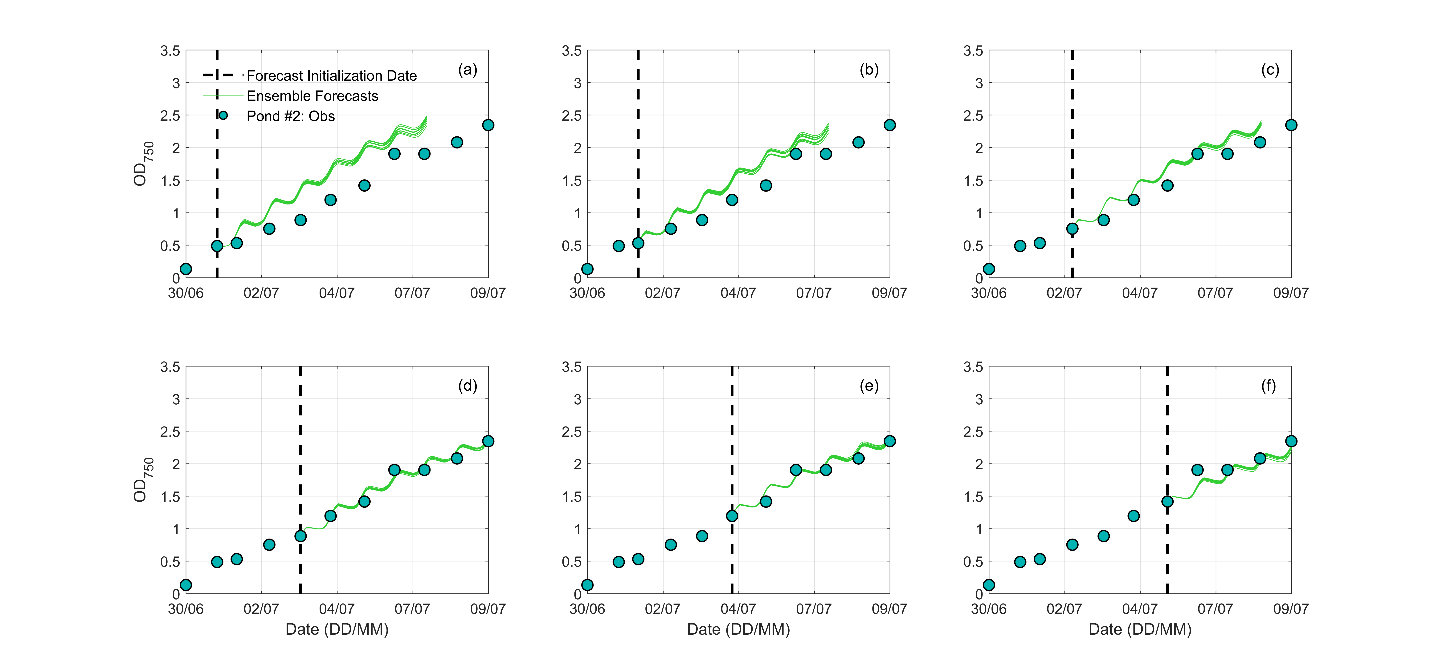


**Figure S5.** Six direct insertion (DI) 7-day microalgae growth forecasts for pond #2.


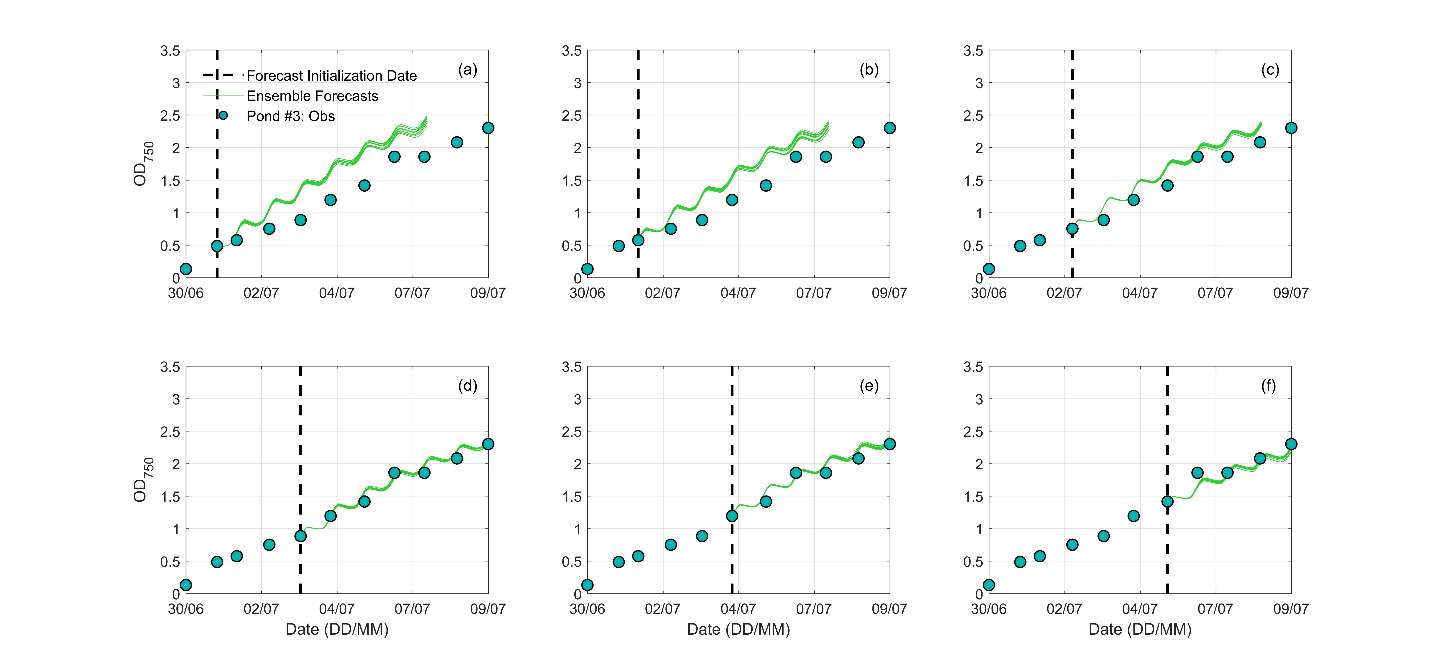


**Figure S6.** Six direct insertion (DI) 7-day microalgae growth forecasts for pond #3.


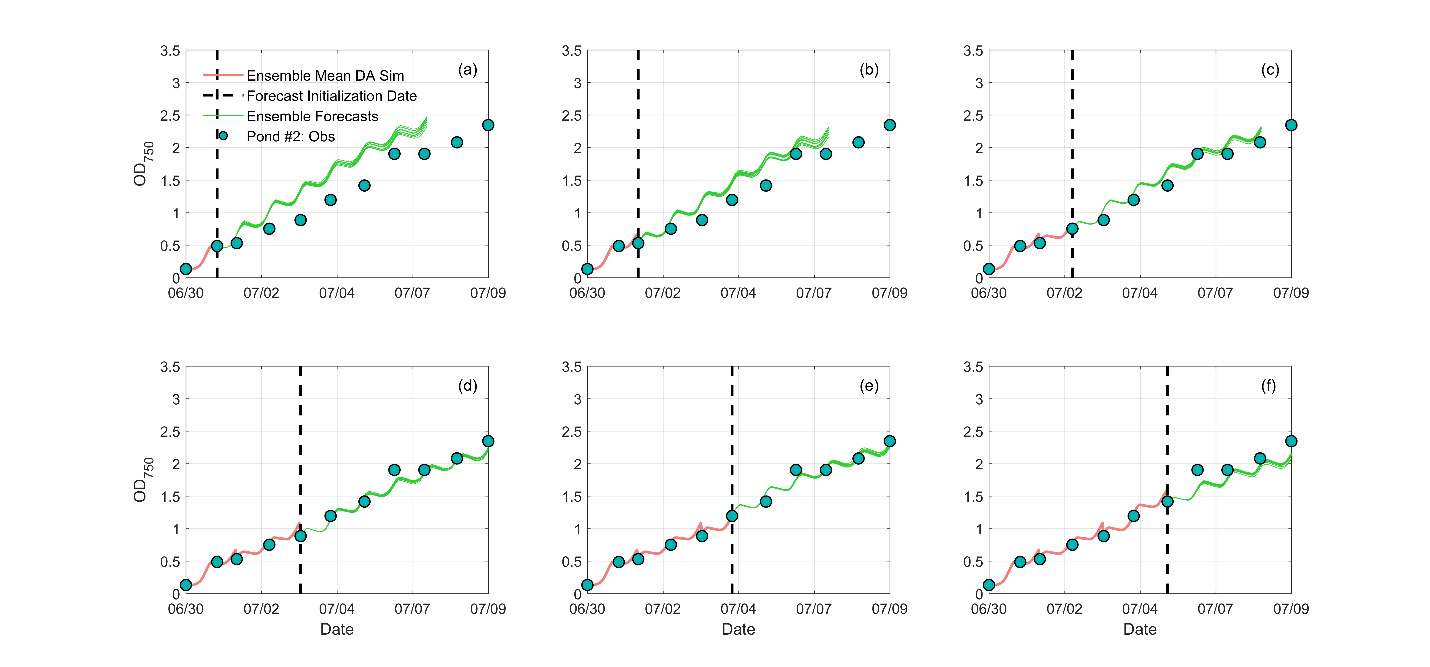


**Figure S7.** Six BGM-DA 7-day microalgae growth forecasts for pond #2.


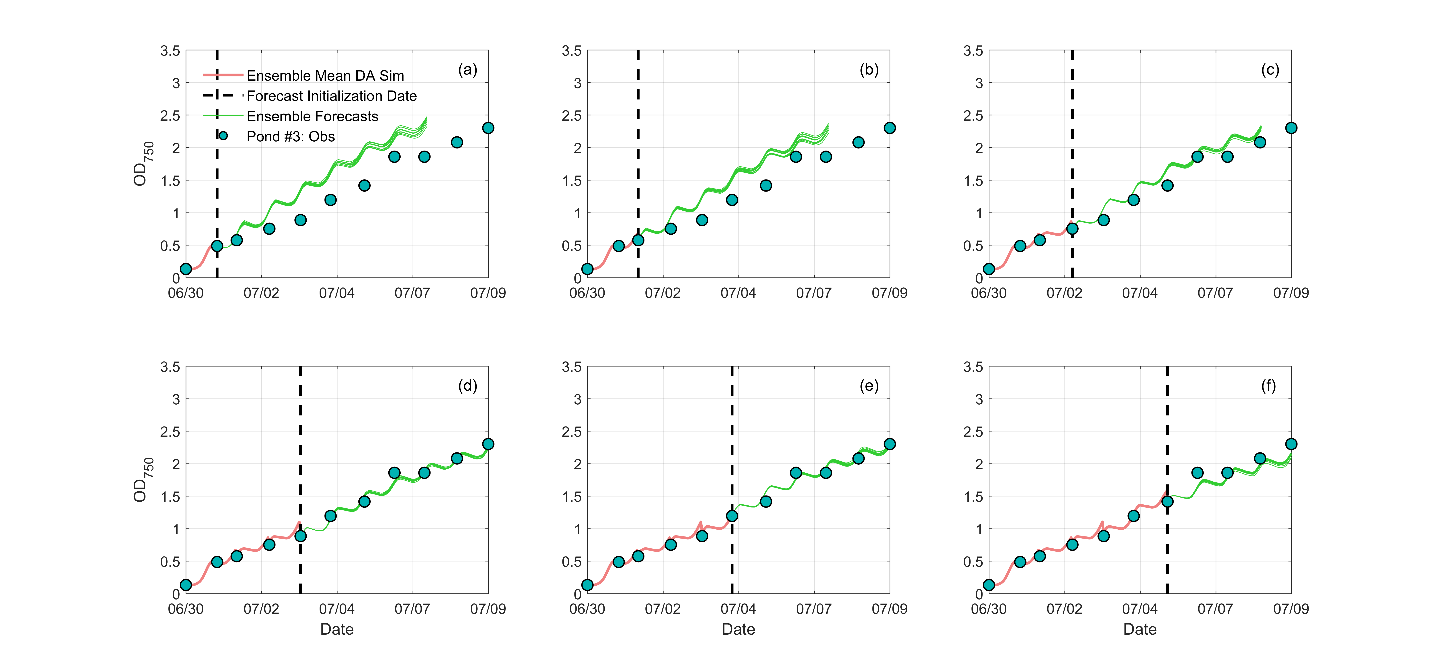


**Figure S8.** Six BGM-DA 7-day microalgae growth forecasts for pond #3.


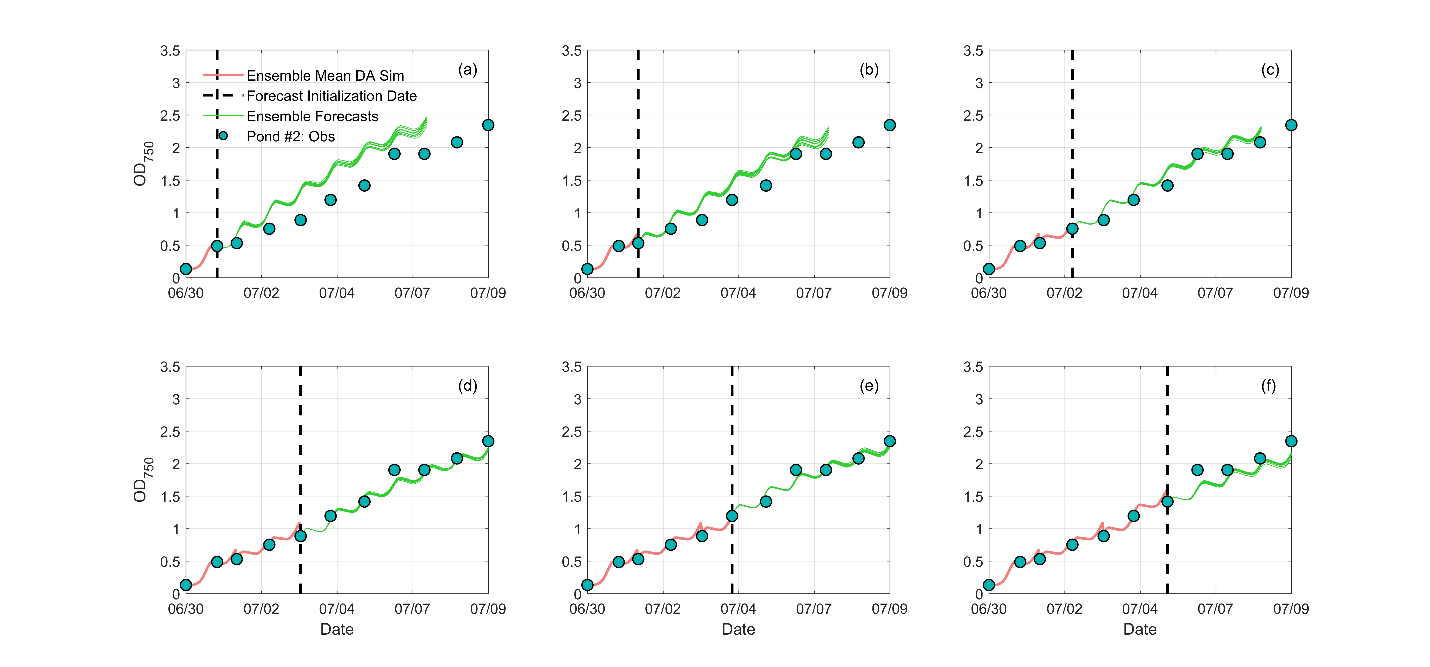


**Figure S9.** Six integrated BGM-MASS2-DA 7-day microalgae growth forecasts for pond #2.


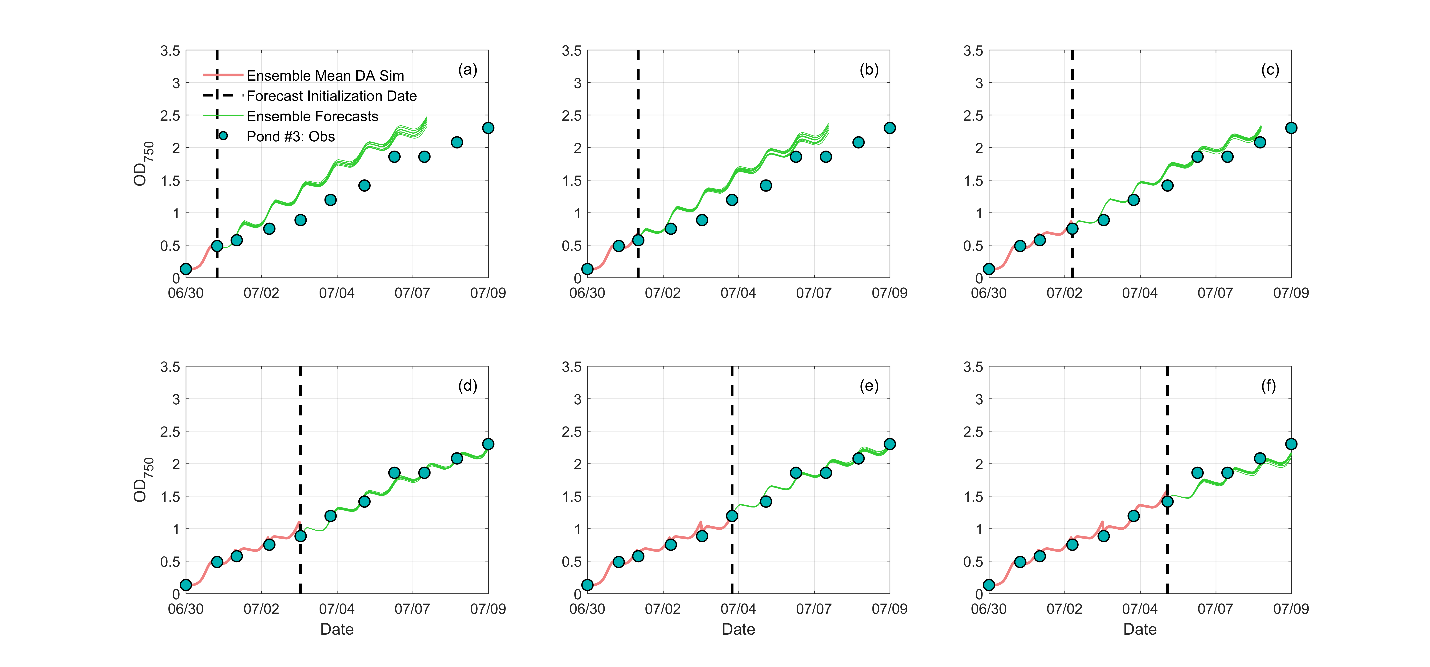


**Figure S10.** Six integrated BGM-MASS2-DA 7-day microalgae growth forecasts for pond #3.
